# Modulated Self-Assembly of Catalytically Active Metal–Organic Nanosheets Containing Zr_6_ Clusters and Dicarboxylate Ligands

**DOI:** 10.1021/acsami.4c00604

**Published:** 2024-04-01

**Authors:** Ram R. R. Prasad, Sophia S. Boyadjieva, Guojun Zhou, Jiangtian Tan, Francesca C. N. Firth, Sanliang Ling, Zhehao Huang, Matthew J. Cliffe, Jonathan A. Foster, Ross S. Forgan

**Affiliations:** † Department of Chemistry, The University of Sheffield, Sheffield S3 7HF, U.K.; ‡ WestCHEM School of Chemistry, University of Glasgow, Joseph Black Building, University Avenue, Glasgow G12 8QQ, U.K.; § School of Chemistry, University of Nottingham, University Park, Nottingham NG7 2RD, U.K.; ∥ Department of Materials and Environmental Chemistry, Stockholm University, Stockholm SE-10691, Sweden; ⊥ Yusuf Hamied Department of Chemistry, University of Cambridge, Cambridge CB2 1EW, U.K.; # Advanced Materials Research Group, Faculty of Engineering, University of Nottingham, University Park, Nottingham NG7 2RD, U.K.

**Keywords:** metal−organic frameworks, coordination modulation, nanosheets, two-dimensional materials, catalysis, organophosphorus compounds, nerve agents, detoxification

## Abstract

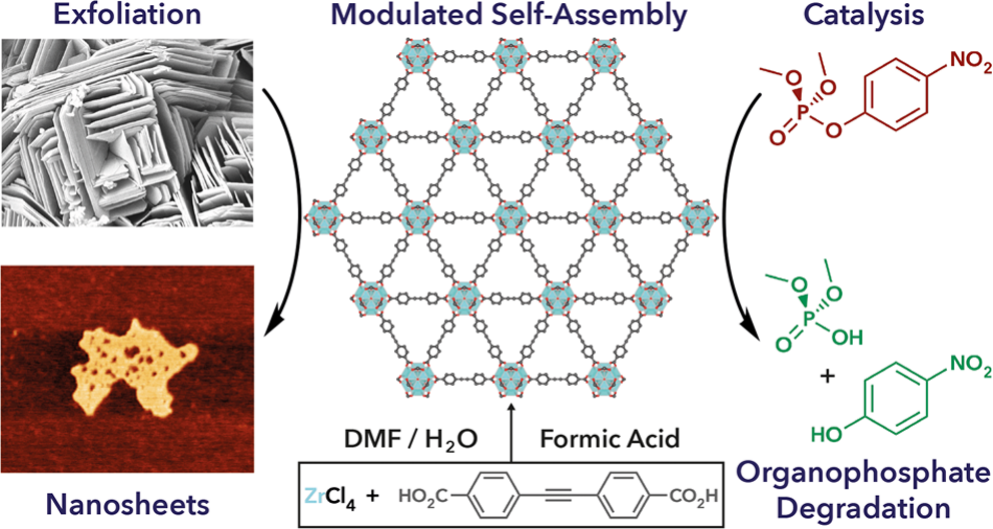

Two-dimensional metal–organic nanosheets (MONs) have emerged as attractive alternatives to their three-dimensional metal–organic framework (MOF) counterparts for heterogeneous catalysis due to their greater external surface areas and higher accessibility of catalytically active sites. Zr MONs are particularly prized because of their chemical stability and high Lewis and Brønsted acidities of the Zr clusters. Herein, we show that careful control over modulated self-assembly and exfoliation conditions allows the isolation of the first example of a two-dimensional nanosheet wherein Zr_6_ clusters are linked by dicarboxylate ligands. The **hxl** topology MOF, termed GUF-14 (GUF = Glasgow University Framework), can be exfoliated into monolayer thickness **hns** topology MONs, and acid-induced removal of capping modulator units yields MONs with enhanced catalytic activity toward the formation of imines and the hydrolysis of an organophosphate nerve agent mimic. The discovery of GUF-14 serves as a valuable example of the undiscovered MOF/MON structural diversity extant in established metal–ligand systems that can be accessed by harnessing the power of modulated self-assembly protocols.

## Introduction

The controlled assembly of metal centers and multitopic organic linkers has been used to great effect in the synthesis of metal–organic nanosheets (MONs) as a versatile class of two-dimensional (2D) materials.^1,2^ In contrast to their three-dimensional (3D) analogues, metal–organic frameworks (MOFs), MONs possess readily tunable surfaces with high external surface area and greater concentration of accessible active sites which makes them ideal candidates for applications in the fields of catalysis, sensing, optoelectronics, and separation.^3−6^ In particular, Zr-based MONs (and their Hf analogues) are of great interest due to their stability under harsh thermal and chemical environments.^7−9^ The high surface area of the nanosheets, the Lewis and Brønsted acidities of the Zr clusters, and the ability to postsynthetically functionalize the surfaces through modulator exchange, have enabled their use in promoting thermocatalytic,^10^ photocatalytic,^11,12^ and electrocatalytic reactions.^13,14^

**Figure 1:**
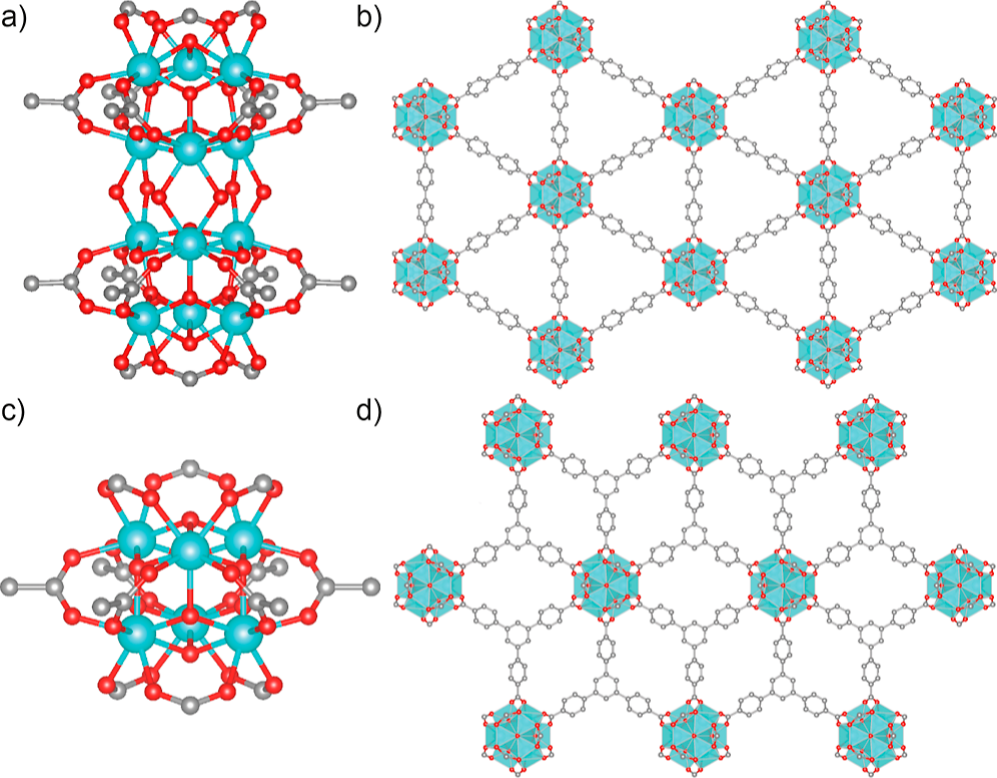
(a) The 12-connected Zr_12_ cluster with six axial capping formates, which connects the 4,4′-biphenyldicarboxylate linkers into (b) hexagonal two-dimensional sheets.^21^ (c) The 6-connected Zr_6_ cluster, with six axial capping formates, that connects 1,3,5-benzenetribenzoate ligands into (d) hexagonal 2D sheets.^8^ Zr: turquoise; C: gray; O: red. H atoms have been omitted for clarity. Zr depicted as ball and stick in (a,c), polyhedral in (b,d). Nanosheets are depicted as single layers viewed down the crystallographic *c* axes.

Combining Zr^4+^ (and/or Hf^4+^) with ditopic carboxylate ligands typically results in 3D MOFs^15^ rather than MONs. For example, the combination of benzene-1,4-dicarboxylate (BDC) with Zr^4+^ can result in the **fcu** topology UiO-66, connected by Zr_6_ clusters;^16^ a related polymorph with **hex** topology;^17^ an **hcp** analogue connected by Zr_12_ clusters (Figure 1a);^18^ and MIL-140A, which has an infinite one-dimensional chain secondary building unit (SBU).^19^ Isolation of 2D phases can be achieved through conversion—either spontaneous or mechanochemically induced—of the **hcp** phase materials into layered, **hxl** topology MOFs (Figure 1b) that can be exfoliated into **hns** topology nanosheets (limited examples can be prepared directly).^20,21^ This means that the preponderance of Zr MONs reported with linear dicarboxylates contains Zr_12_ clusters.

In contrast, while the combination of Zr^4+^ and tricarboxylic acids can give 3D structures such as MOF-808,^22^ other syntheses with tritopic linkers using modulated^23^ solvothermal conditions can *directly* lead to 2D systems, typically containing Zr_6_ clusters (Figure 1c), that can be exfoliated into MONs (Figure 1d).^8,24,25^ Other relevant systems include tricarboxylate linked Hf_12_ clusters reported by Lin and co-workers, in which the extended 4,4′,4″-[benzene-1,3,5-triyl-tris(ethyne-2,1-diyl)]tribenzoate linker bridges 12-connected Hf_12_ clusters.^26^ Wang and co-workers reported Zr-NiTCPP, where the Zr_6_ clusters are linked by tetratopic nickel-tetrakis(4-carboxyphenyl)-porphyrin.^27^ Stock and co-workers reported CAU-45, possessing both Zr_6_ and Zr_12_ clusters linked by the 5-acetamidoisophthalate linker, which could be exfoliated by ultrasound-assisted liquid-phase exfoliation to access MONs.^28,29^ A 2D layered structure where Zr_6_ clusters are connected entirely by formate ligands has also been reported.^30^

Whether nanosheets are formed directly from solution (bottom-up) or by exfoliating the layered materials using ultrasound energy to overcome the weak interlayer interactions (top-down), it is clear that judicious control of reaction conditions, including the use of modulators, is necessary to generate phase-pure MONs as opposed to 3D counterparts. We have recently explored^31^ the phase space in Zr^4+^ MOFs of the 4,4′-(ethyne-1,2-diyl)dibenzoate (EDB^2–^) ligand, where the combined use of acetic acid as modulator and controlled amounts of water can favor the formation of an **hcp** topology phase with Zr_12_ clusters [GUF-12(Zr)] over the more commonly observed **fcu** phase^32,33^ with Zr_6_ clusters. Building upon these investigations, we have now isolated an alternative phase by using specific quantities of formic acid as the modulator. We have shown by 3D electron diffraction that this phase comprises a 2D material with **hxl** topology—an unusual example of a 2D MOF containing linear dicarboxylate ligands and Zr_6_ clusters—that we have named GUF-14. Herein we describe the isolation and characterization of GUF-14, its exfoliation into MONs, and their catalytic activity.

## Results and Discussion

**Figure 2:**
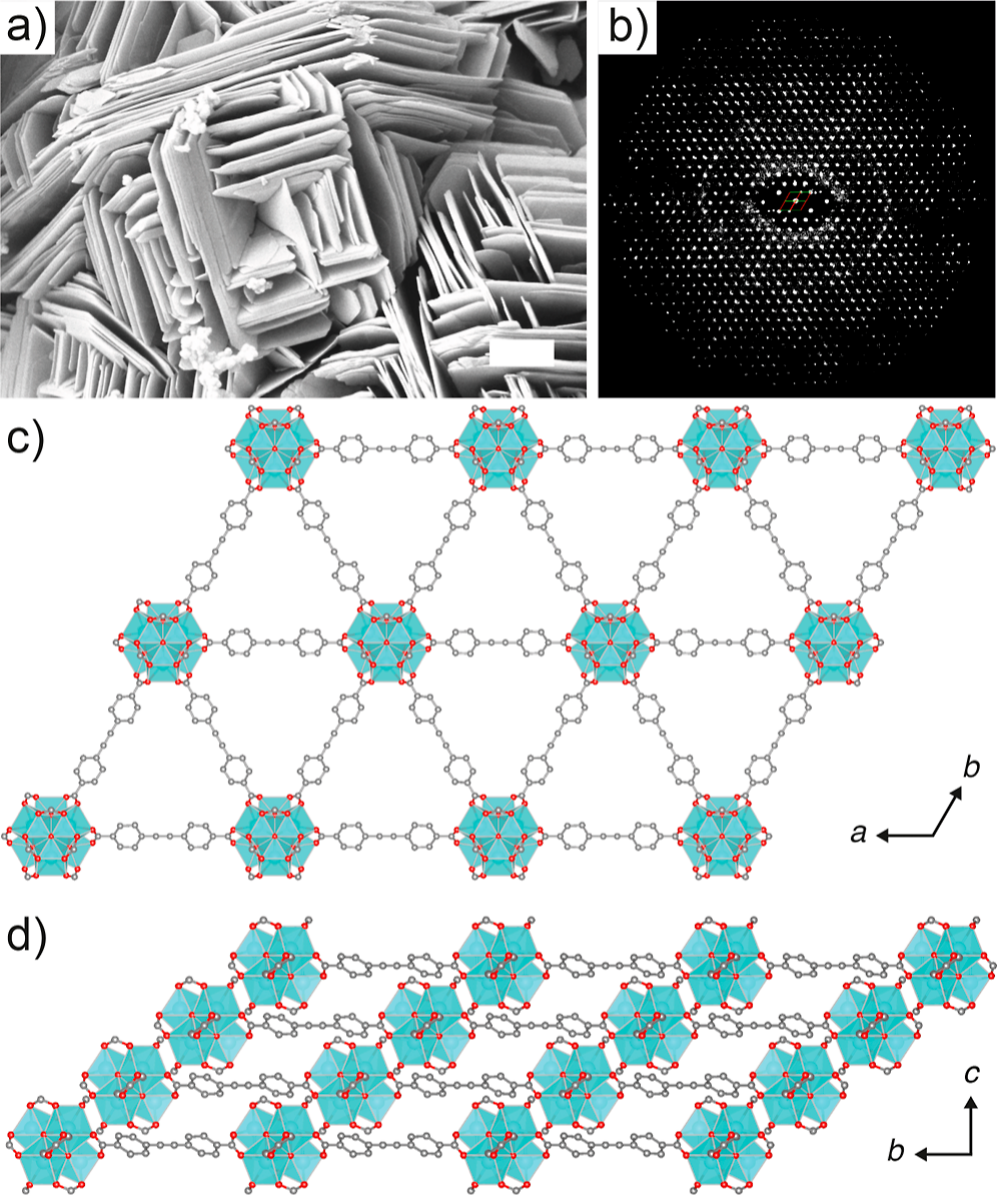
(a) Scanning electron micrograph of GUF-14 (scale bar 1 μm). (b) 2D slice cut from the 3D reciprocal lattice of GUF-14, reconstructed from cRED data, showing the *hki*1 plane. (c) The hexagonal structure of one layer of GUF-14 viewed down the crystallographic *c* axis. (d) ABC packing arrangement of these layers in GUF-14 as viewed down the crystallographic *a* axis. Partial packing structures are derived from cRED data. C: gray; O: red; Zr: turquoise polyhedra. H atoms omitted for clarity. Formate capping ligands depicted as fully occupied.

We first reported the **fcu** phase of Zr-EDB, which has formula [Zr_6_O_4_(OH)_4_(EDB)_6_], in 2015, where the use of l-proline as modulator allowed access to large single crystals^32^ (at the same time the material was independently reported by a different group and denoted as BUT-30^33^). As part of our ongoing interest in drug delivery from Zr MOFs,^34^ we attempted to prepare nanoparticles of this phase by modulated self-assembly, discovering the **hcp** phase, GUF-12(Zr), when using 100–120 equiv acetic acid and 0.5–2.5% (*v/v*) water in solvothermal syntheses in *N*,*N*-dimethylformamide (DMF) at 423 K.^31^ This material, with ideal formula [Zr_12_O_8_(OH)_14_(EDB)_9_], was found to be defective, but it could not be converted into a 2D material as has been achieved with isoreticular derivatives.^20,21^ Formic acid is also a potent modulator in Zr MOF synthesis: it has a lower p*K*_a_ than acetic acid and so has greater potential for decreasing particle size^35^ and inducing defectivity.^36^ Replicating the previous conditions with 105–130 equiv of formic acid as modulator and 0.5% (*v/v*) water in DMF solvothermal syntheses at 423 K generated a new phase, whose structure we could not determine ab initio by powder X-ray diffraction (PXRD), but which showed a clearly different morphology by scanning electron microscopy (Figure 2a).

To identify the material, we turned to continuous rotation electron diffraction (cRED, Figure 2b, Section S2),^37−40^ which revealed a new 2D-layered Zr MOF, denoted GUF-14, with trigonal space group *R*3̅*m* and unit cell dimensions *a* = 21.007(3) and *c* = 14.647(3) Å. In contrast to the 18- and 12-connected dodecanuclear Zr_12_ clusters observed in the **hcp** and **hxl** topology Zr-dicarboxylate systems,^21^ GUF-14 comprises 6-connected Zr_6_ SBUs, with 8-coordinate Zr^4+^ ions linking EDB^2–^ ligands into a network with **hxl** topology (Figure 2c). The 2D, hexagonal sheets adopt an ABC-layer stacking arrangement, where individual layers shift one-third of unit cell along the *ab* direction, and two-thirds along the *a* + 1/2*b* direction (Figure 2d).

In contrast to the 6-connected SBU found in the 3D MOF-808,^22^ where the carboxylate ligands are found in triads at opposite faces of the SBU, the carboxylate ligands in GUF-14 are found in the inverse arrangement, in sites around the equator of the cluster, leading to the hexagonal topology. An analogous SBU has previously been observed in the 2D [Hf_6_O_4_(OH)_4_(HCO_2_)_6_(BTB)_2_] material (BTB = benzene-1,3,5-tribenzoate),^8,24,25^ with Zr homologues reported (Figure 1c).^41^ In these examples, six formate ligands complete the coordination at the SBU—formate is well-known to stabilize lower connectivity Zr_6_ SBUs through incorporation as a capping ligand^42^—but in this work only a small quantity of formate in GUF-14 could be visualized by cRED. The occupancy of the formate carbon position in the asymmetric unit was freely refined to 0.28(16); as the oxygens around the cluster are fully occupied, we can assume a disorder model encompassing the formate and a pair of hydroxide and water ligands,^43,44^ giving an overall formula for the cRED structure of GUF-14 of [Zr_6_(μ_3_-O)_4_(μ_3_-OH)_4_(OH)_4.3_(H_2_O)_4.3_(HCO_2_)_1.7_(EDB)_3_]. A structurally related material—CAU-26—has been reported as a kinetic product in the reaction of zirconium acetate with benzene-1,4-dicarboxylic acid in neat acetic acid. The material, formulated as [Zr_6_O_4_(OH)_4_(OAc)_6_(BDC)_3_], exhibited low crystallinity and high defectivity, but electron diffraction data suggested a layered structure with **hxl** topology with eclipsed AA stacking and a larger interlayer distance.^45^ Similarly, the reaction of ZrOCl_2_ with 3,3″,5,5″-tetramercapto[1,1′:4′,1″-terphenyl]-4,4″-dicarboxylic acid in DMF and formic acid led to a material, Zr-TPDCS-2, which was inferred to have an isoreticular layered structure by PXRD analysis.^46^ In both cases, a structure was not refined from the diffraction data.

**Figure 3:**
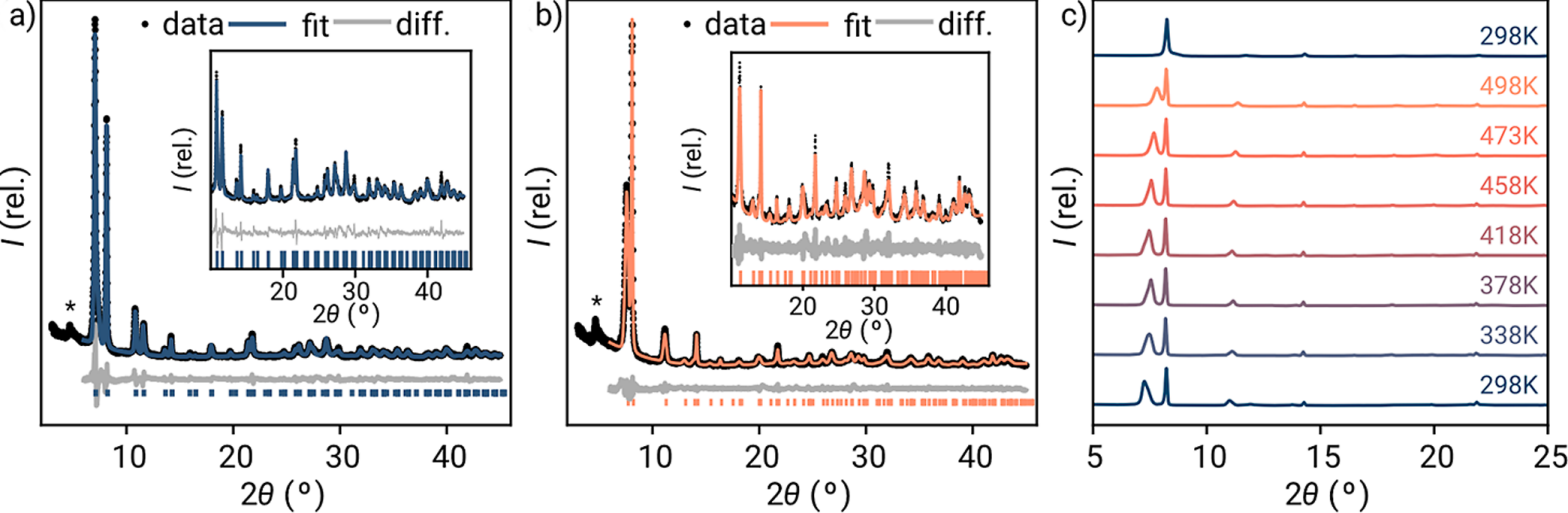
Pawley fitting of the powder X-ray diffractograms of GUF-14 in (a) the as-synthesized form and (b) after activation under vacuum at 423 K for 16 h. (c) Variable temperature powder X-ray diffractograms recorded on an activated sample of GUF-14, sequentially from bottom to top.

Synthetic optimization (Section S3.1) revealed that, for bulk syntheses, 125 equiv of formic acid and 0.5% (*v/v*) water in DMF reliably yields as-synthesized samples of GUF-14, with powder X-ray diffractograms closely resembling those predicted from the structure elucidated by electron diffraction. Pawley refinement (Figure 3a) of the experimental powder diffraction data in the space group determined via cRED provided a good fit, with refined lattice parameters *a* = 21.466(4) Å and *c* = 16.666(4) Å consistent with those determined from cRED but with a longer *c* axis. We hypothesize that interlayer contraction along the *c* axis occurs as solvent is lost in the vacuum chamber of the transmission electron microscope, leading to this difference with the as-synthesized samples of GUF-14. An additional feature, consisting of a peak with an extremely asymmetric tail toward high angle, was present at low angle (2θ ∼ 4.5°) but is not accounted for by the *R*3®*m* space group (marked with an asterisk in Figure 3). The peak position corresponds to the (001) Bragg position, which would be systematically forbidden in *R*3®*m*, and the Warren-like peak shape suggests that it arises from the presence of stacking faults between layers.^47^ To assess the phase purity of the as-synthesized GUF-14 material, density functional theory (DFT) calculations were employed (Section S3.2) to generate a structural model of as-synthesized GUF-14 based upon a fully optimized DFT structure of GUF-14 with formate as the capping unit, but partially re-optimized with a fixed *c* axis parameter of 16.66 Å, derived from the Pawley fit in Figure 3a. Comparison of the predicted powder X-ray diffractogram for this DFT model structure with the experimental data for as-synthesized GUF-14 showed an excellent structural match and confirmed overall phase purity (Figures S7 and S13).

Acid digestion of a sample of GUF-14 that had been washed with acetone and dried under a turbo-pump vacuum at 393 K for 20 h allowed ^1^H nuclear magnetic resonance (NMR) spectroscopic analysis to determine the level of formate inclusion in the bulk structure (Section S3.3). A fully formate capped Zr_6_ SBU would yield a MOF with the ideal formula [Zr_6_(μ_3_-O)_4_(μ_3_-OH)_4_(HCO_2_)_6_(EDB)_3_] and an EDB^2–^/HCOO^–^ ratio of 1:2, but ^1^H NMR spectra of acid-digested samples gave an EDB^2–^/HCOO^–^ ratio of 1:1.1, higher than that observed by cRED (1:0.6) but still short of a fully formate capped structure. As such, the bulk material likely corresponds to a partially formate-capped material with—assuming that water and hydroxide (in the absence of any other NMR spectroscopically visible organic capping ligands) comprise the residual capping species^43,44^—approximate formula [Zr_6_(μ_3_-O)_4_(μ_3_-OH)_4_(HCO_2_)_3.3_(OH)_2.7_(OH_2_)_2.7_(EDB)_3_], but with particle-to-particle variations. Thermogravimetric analysis (TGA) of the activated sample in air showed a three-step mass loss process, with the final residue (41.3 wt % at 1053 K) corresponding well to that calculated from the formula derived by NMR spectroscopy (43.1 wt % assuming the residue is ZrO_2_).

The capping formates of the Zr_6_ cluster and the alkyne spacer of the adjacent linker are separated by only 3.8 Å in the cRED structure. This short distance likely explains why formic acid modulation yields GUF-14 with 6-connected Zr_6_ SBUs and **hxl** topology, while identical syntheses using acetic acid as modulator yield GUF-12, with 18-connected Zr_12_ SBUs and **hcp** topology; modulator capping is required to yield the 6-connected SBU, but the close-packing arrangement precludes the bulkier acetic acid capping the SBU. Indeed, syntheses employing larger modulating agents such as trifluoroacetic acid, benzoic acid, or 3-fluorobenzoic acid (which has a p*K*_a_ value close to formic acid) did not yield GUF-14 (Supporting Information, Section S4).^48^ Similarly, attempts to prepare isoreticular versions of GUF-14 using biphenyl-4,4′-dicarboxylate linkers under the optimized conditions resulted in the formation of UiO-67 phases with **fcu** topology, highlighting the need for the alkyne spacer to facilitate this particular structure. Synthetic attempts using the shorter acetylenedicarboxylate linker were also unsuccessful, possibly due to the thermal instability of the ligand. These results further imply that the minimal steric profiles of the alkyne spacer and the small formate capping units are essential to provide enough room for the formate caps of Zr_6_ clusters to sit between the layers and allow formation of the **hxl** phase. The low steric profile of the alkyne moiety has previously allowed isolation of other unexpected MOF phases linked by different metals.^49−51^

After removal of residual solvents, by heating, drying under vacuum, or a combination of both, changes in the powder X-ray diffractograms of GUF-14 were evident. For example, the diffractogram of a sample of GUF-14 that had been heated at 423 K for 16 h in a programmable oven shows shifting and broadening of the Bragg peak originally located around 7° 2θ—the (101) reflection—in the as-synthesized material. Pawley refinement (Figure 3b) of this desolvated sample in the *R*3®*m* space group revealed that this shift corresponds to a significant *c* axis contraction to 14.486(6) Å; this 13% contraction gives a *c* axis parameter shorter than that observed by cRED, and is accompanied by a small but significant expansion (0.2%) of the *a* axis to 21.516(2) Å. We were able to qualitatively reproduce the experimental pattern using models derived from DFT as a starting point (Figure S13), though significant stacking faults, the presence of unresolved guests, and preferred orientation prevented full quantitative refinement.

To explore the origin of this structural change, we carried out further DFT calculations on GUF-14 modeled with different capping units (Section S3.2). Our DFT calculations, which were based on a more ordered distribution of capping ligand, being either formate, hydroxide, or hydroxide with water, show that the interlayer spacing, which determines the lattice parameter along the *c* axis, is very sensitive to the identity of the capping unit. We found that replacing formate by hydroxide in these models led to a significant contraction in the *c* axis, from 14.20 to 12.88 Å (10%), of comparable magnitude to that found on desolvation of the experimental GUF-14. We note the experimentally determined lattice parameter along the *c* axis for the desolvated sample is 14.486 Å, which is a result of spatial averaging from the Pawley refinement, and there may be local regions in the sample with bigger or smaller interlayer spacing, i.e., closer to what we see in our DFT calculations with hydroxide or hydroxide/water as a capping unit. Additional DFT calculations of the alternative “AB” type stacking show that the two stacking sequences are comparable in energy, with the energy difference depending strongly on the identities of both capping ligands and guests (*E*_AB_ – *E*_ABC_ = −10.6 kJ mol^–1^ per Zr_6_ cluster for formate capping, −52.9 kJ mol^–1^ per Zr_6_ cluster for hydroxide capping, and +91.1 kJ mol^–1^ per Zr_6_ cluster for hydroxide capping with guest water), providing further evidence for the feasibility of the stacking faults postulated from PXRD analysis.

Further exploring the structural changes observed upon heating, variable temperature PXRD analysis of GUF-14 (Figure 3c) shows a gradual decline in the intensity of the (101) Bragg peak, eventually resulting in its disappearance when heated at 498 K for 16 h, together with all other peaks of mixed *hkl* character. This transition is irreversible—cooling to room temperature does not indicate any structural reversion—and is attributed to the delamination of the stacked layers seen in scanning electron micrographs, resulting in the transition to a **hns** phase.

**Figure 4:**
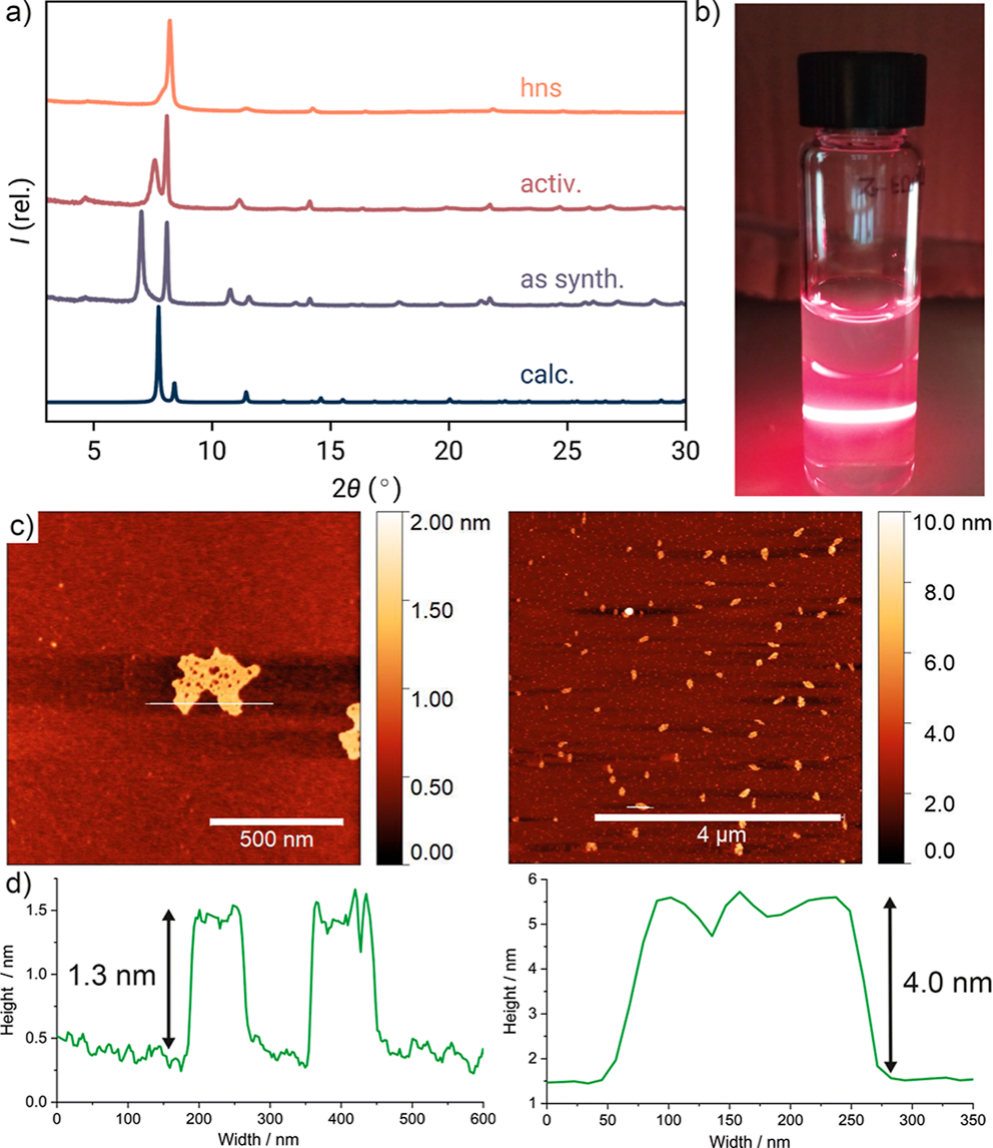
(a) Stacked partial powder X-ray diffractograms of GUF-14 **hxl** as-synthesized (as synth.), after activation (activ.), and after delamination to the GUF-14 nanosheet phase (hns) compared with the pattern predicted from the cRED structure of GUF-14. (b) Tyndall scattering effects exhibited by as-prepared suspensions of GUF-14 **hns** in water. (c) Atomic force microscope (AFM) topographic images of GUF-14 **hns** in water (left) and ethanol (right) together with (d) their corresponding height profiles.

To further investigate this delamination into MONs, ultrasound-assisted liquid-phase exfoliation of bulk GUF-14 (**hxl**) was performed at 37 kHz for 12 h in a range of different solvents (Section S5). The obtained nanosheets were then isolated from suspensions by centrifugation at 1500 rpm for 1 h to remove unexfoliated MOF and larger fragments. As partial delamination was observed during heating, GUF-14 **hxl** was first activated at 473 K for 16 h to aid the transition toward the **hns** phase, prior to ultrasound-assisted liquid-phase exfoliation which could be observed by PXRD (Figure 4a).

Optimization of the process showed exfoliation in water and ethanol to be more efficient than in acetonitrile, as evidenced by Tyndall scattering (Figure 4b). Atomic force microscopy (AFM, Figure 4c,d) showed that water-based exfoliation produced thinner MONs of GUF-14, down to 1.3 nm, which is comparable to the van der Waals diameter of a single monolayer of Zr_6_ clusters, and so the following description of the GUF-14 **hns** phase refers to samples exfoliated in water. The stability and high degree of crystallinity of the GUF-14 nanosheets were demonstrated by the powder X-ray diffractogram collected on a sample isolated by centrifugation at 4500 rpm for 2 h (Figure 4a).

Removal of monotopic “capping” carboxylate units at MOF/MON secondary building units can generate Lewis^52,53^ and Brønsted^54^ acidic sites capable of promoting organic transformations.^55^ As the GUF-14 **hxl** and **hns** phases contain Zr_6_ clusters with potentially labile coordinated formate groups, we hypothesized that acid washing and subsequent activation could generate catalytically active acidic sites.^56^ Following the protocol reported by Stoddart and co-workers, samples of GUF-14 as the **hxl** and **hns** phases were washed with aqueous 1 M HCl (Section S6.1).^57^ The materials obtained were collected by centrifugation and activated at 423 K, with ^1^H NMR spectra of NaOD digests showing loss of >80% of capping formates while no structural degradation was observed by PXRD. Their catalytic activities toward the imine condensation reaction between 4′-fluoroacetophenone and benzylamine in toluene were assessed and compared with those of pristine samples of the Zr-EDB MOF in the **fcu** topology (Section S6.2). After 24 h, ^19^F{^1^H} NMR spectroscopy (Table 1) showed that the exfoliated MONs—the GUF-14 **hns** phase—induced a much higher conversion toward the desired imine (78% by ^19^F{^1^H} NMR spectroscopy; isolated yield 71%) compared to both GUF-14 **hxl** (14%), Zr-EDB **fcu** (22%), and an uncatalyzed control (17%; Table 1, entries 1–4). It would be expected that the GUF-14 **hns** and **hxl** phases, with 6-connected Zr_6_ SBUs and potentially half of all carboxylate coordination sites available for catalysis, show higher activity than the Zr-EDB **fcu** phase, where the only feasible equivalent active sites would be at particle surfaces or missing linker defects around the 12-connected Zr_6_ SBU. The much lower conversion observed for the bulk GUF-14 **hxl** phase in comparison to the GUF-14 **hns** nanosheets demonstrates that reagent accessibility is key.

**Table 1:**
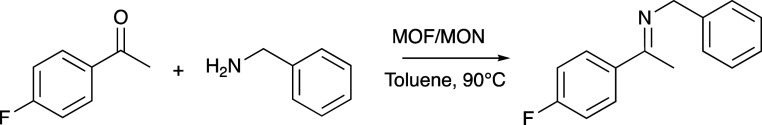
Imine Condensation between 4′-Fluoroacetophenone and Benzylamine^a^

entry	catalyst^a^	product (%)^b^
1	no catalyst	17
2	Zr-EDB **fcu**	22
3	GUF-14 **hxl**	14
4	GUF-14 **hns**	78
5^c^	GUF-14 **hns** (recycled)	75
6^d^	GUF-14 **hns** (recycled ×2)	75
7^e^	GUF-14 **hns** (5 h, filtered)	26
8^f^	GUF-14 **hns** (filtered, +19 h)	25

a Reaction conditions: 4′-fluoroacetophenone (1 mmol), benzylamine (1.3 mmol), 1-methylnaphthalene (0.5 mmol) and activated catalyst (1 mol % of Zr-EDB **fcu** or 0.5 mol % of acid washed GUF-14 **hxl** or **hns**) added to 3 mL of toluene and heated at 363 K for 24 h.

b Conversion determined by ^19^F{^1^H} NMR spectroscopy.

c Mol % of recovered GUF-14 **hns** restored to 0.5 mmol % with 0.5–0.9 mg fresh or recycled catalyst to account for material lost during centrifugation, washing and activation procedure.

d The recovery process was repeated a second time.

e Reaction stopped after 5 h and filtered.

f Filtered supernatant reacted for further 19 h.

To demonstrate the recyclable nature of the GUF-14 **hns** nanosheets, they were collected via centrifugation post-catalysis, washed multiple times with toluene and acetone, and then activated at 423 K, prior to their reuse in catalyzing the same reaction. The recycled MONs induced 75% conversion to the imine in both the second and third reactions, confirming their reusable nature (Table 1, entries 5 and 6). The GUF-14 **hns** MONs recovered after catalysis show retention of crystallinity and morphology, as evidenced by PXRD and AFM. The heterogeneous nature of the reaction was confirmed by a filtration test where, after 5 h reaction, the supernatant was carefully removed using a syringe fitted with 0.1 μm PES filter, transferred to another vial, and then continued to be heated under standard reaction conditions. No further conversion was observed after removal of the catalyst, proving the heterogeneous nature of the catalysis (Table 1, entries 7 and 8).

**Figure 5:**
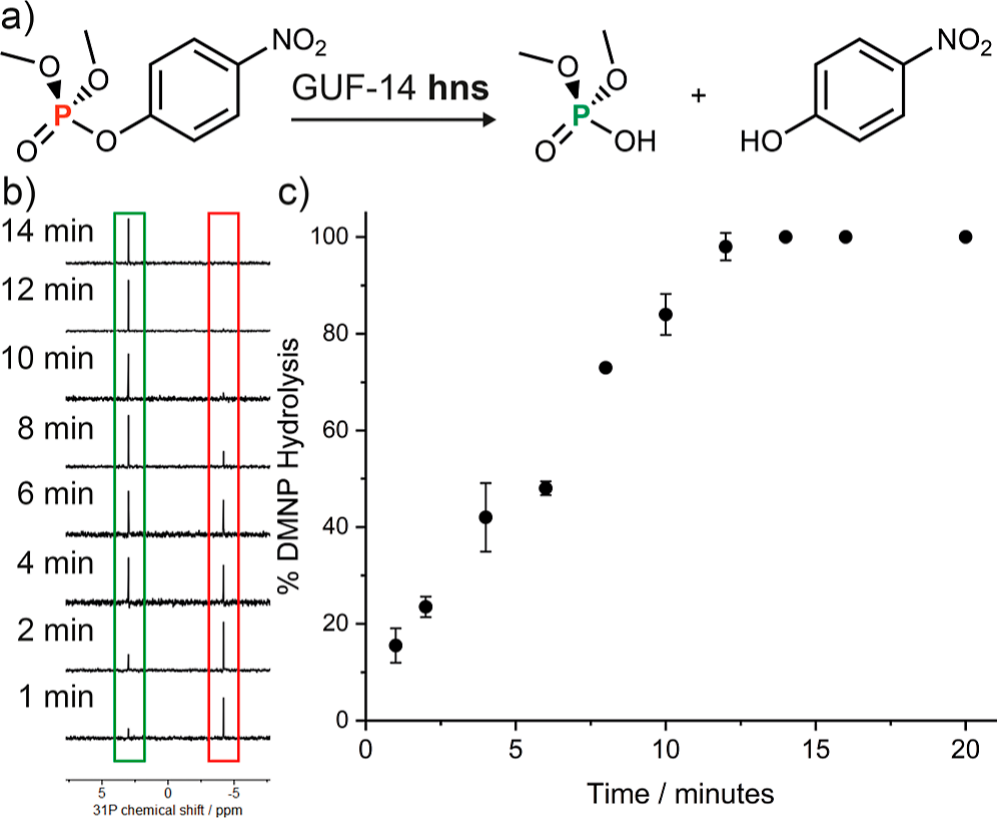
GUF-14 **hns** catalyzed hydrolysis of nerve agent simulant DMNP. (a) Reaction scheme of DMNP hydrolysis. (b) Partial ^31^P{^1^H} NMR spectra showing time dependent DMNP hydrolysis. (c) Averaged data from the two replicate catalytic reactions with standard deviation included as error bars.

The enhanced catalytic performance of the GUF-14 **hns** nanosheets in comparison to the bulk **hxl** material and its 3D **fcu** topology counterpart inspired us to further examine the catalytic activity of the MONs in the hydrolysis of the nerve agent simulant dimethyl (4-nitrophenyl)phosphate (DMNP, Figure 5a). Other Zr MOFs/MONs with low connectivity clusters are known to exhibit exceptional activity for this reaction and related conversions.^58,59^ The hydrolysis reaction was carried out via the procedure reported by Farha and co-workers, with ^31^P{^1^H} NMR spectroscopy used to assess conversion (Section S6.3).^60^

The GUF-14 **hns** MONs were able to completely hydrolyze DMNP within 12 min (Figure 5b,c), with a *t*_1/2_ of ∼6.5 min, outperforming several other known Zr_6_ cluster-based MOFs such as PCN-222, NU-1000 and UiO-66,^59^ again indicative of the high accessibility of the coordinatively unsaturated Zr_6_ cluster. However, the *t*_1/2_ of the GUF-14 **hns** phase is higher than those reported for the Zr-BTB MON (*t*_1/2_ = 2.1 min)^61,62^ and the benchmark catalyst MOF-808(Zr) (*t*_1/2_ = <0.5 min),^58^ suggesting that further optimization of structure or activation would be required to closely compete with sector-leading materials.

## Conclusions

In conclusion, we show that careful control of modulated synthetic conditions can lead to the isolation of valuable new MOF phases—in particular, MONs—in established metal–ligand systems. The use of an extended alkyne linker and formic acid as a modulator is key to the discovery of GUF-14, a 2D-layered MOF in which Zr_6_ clusters are connected by dicarboxylate linkers with **hxl** topology, as they allow close packing between layers in an unusual ABC stacking arrangement, in comparison to the previously reported GUF-12 which has **hcp** topology and Zr_12_ clusters. Activation at high temperatures results in the **hxl** topology of GUF-14 compressing along the *c* axis which, following ultrasound-assisted liquid-phase exfoliation, undergoes a phase transition to form GUF-14 MONs, with **hns** topology, via delamination of the stacked layers. Acid washing to remove coordinated formate units enhances the catalytic activity of GUF-14 MONs compared to their 3D- and 2D-layered counterparts, leading to a material with excellent kinetics for hydrolysis of an organophosphate nerve agent mimic. We anticipate that these new members of the Zr MOF/MON families, capable of forming monolayer nanosheets with catalytic functionalities, will prove useful in a wide range of catalysis, sensing, water-purification, and gas-separation applications, while serving as examples of the power of modulated self-assembly in the discovery of novel, functional, network solid materials.

## Experimental Section

### Materials

All solvents and reagents were purchased from chemical suppliers and used without further purification. Methyl 4-iodobenzoate (99%) and trimethylsilylacetylene (99%) were purchased from fluorochem. 1,8-Diazabicyclo[5.4.0]undec-7-ene (DBU) (98%) and bis(triphenylphosphine)palladium(II) dichloride were purchased from Sigma-Aldrich (Merck). Triethylamine (99%), formic acid (97%) and glacial acetic acid (99+%) were obtained from Alfa Aesar and zirconium chloride (ZrCl_4_) (98%) was purchased from Acros Organics. The linker precursor dimethyl 4,4′-(ethyne-1,2-diyl)dibenzoate and the linker 4,4′-(ethyne-1,2-diyl)dibenzoic acid (EDB-H_2_) were synthesized according to our reported synthesis procedures.^32^ NMR spectroscopic data (Figures S1 and S2) are in accordance with literature reports.^63^ Zr-EDB **fcu** was synthesized according to our own reported synthesis procedure.^32^ The powder X-ray diffractogram matched that predicted from the single-crystal structure (Figure S3).

### Powder X-ray Diffraction

Powder X-ray diffraction (PXRD) analysis was carried out using a Bruker-AXS D8 diffractometer with primary monochromation (Cu Kα1, λ = 1.5418 Å) and a LynxEye position sensitive detector in Bragg–Brentano parafocusing geometry using either 0.7 mm quartz glass capillaries or a or silicon low background sample holder, or a PANalytical X’Pert PRO diffractometer (λ(Cu Kα) = 1.54056 Å) with a mounted bracket sample stage. Variable temperature-PXRD (VT-PXRD) measurements were collected on the Bruker-AXS D8 diffractometer in air, with a ramp rate of 5 K min^–1^, and held at the desired temperature for 30 min prior to measurements. Data were collected on heating from 298 to 498 K and after cooling down to 298 K using a Cobra Plus nonliquid-nitrogen cryostream (Oxford Cryosystems).

### Continuous Rotation Electron Diffraction

Continuous rotation electron diffraction (cRED) samples were dispersed in acetone, and a droplet of the suspension was transferred onto a carbon-coated copper grid. Observation was performed on a JEOL JEM2100 microscope operated at 200 kV (Cs = 1.0 mm, point resolution = 0.23 nm). Images were recorded with a Gatan Orius 833 CCD camera (resolution 2048 × 2048 pixels, pixel size 7.4 μm) under low dose conditions. Electron diffraction patterns were recorded with a Timepix pixel detector QTPX-262k (512 × 512 pixels, pixel size 55 μm, Amsterdam Sci. Ins.). The data were collected using the software Instamatic.^37^ A single-tilt tomography holder was used for the data collection, which could tilt from −70 to +70° in the microscope. The aperture used for cRED data collection was about 1.0 μm in diameter. The speed of the goniometer tilt was 0.45° s^–1^, and the exposure time was 0.5 s per frame. Three data sets were collected within 3.4, 3.5, and 3.7 min to minimize the beam damage and to maximize the data quality. The covered tilt angles are 91.71, 94.56, and 99.95°, respectively. In order to increase the completeness, these data sets were merged via using XDS package.^64^

### Nuclear Magnetic Resonance

Nuclear magnetic resonance spectra were recorded on either a Bruker AVIII 400 MHz spectrometer or a Bruker Avance III HD 400 spectrometer and referenced to residual solvent peaks. Samples of bulk GUF-14 were prepared for analysis by digestion in DMSO-*d*_6_/D_2_SO_4_. Samples of exfoliated GUF-14 nanosheets were prepared by dissolving 10–15 mg in 1 mL of 1 M NaOH in D_2_O by vigorous stirring for 24 h and filtered through cotton wool to remove solid particles.

### Thermogravimetric Analysis

Thermogravimetric analysis (TGA) measurements were carried out using a TA Instruments Q500 Thermogravimetric Analyzer. Measurements were collected from room temperature to 1073 K with a heating rate of 10 K min^–1^ in air.

### Scanning Electron Micrographs

Scanning electron micrographs were obtained using a Carl Zeiss Sigma Variable Pressure Analytical SEM with Oxford Microanalysis after the powder samples were coated with Pd for 50 s using Polaron SC7640 sputter coater.

### Atomic Force Micrographs

Atomic force micrographs were collected using a Bruker Multimode 5 atomic force microscope (AFM) fitted with a Nokia 10× visualizing lens operating in soft-tapping mode in air under ambient conditions. Bruker OTESPA-R3 cantilever with 20.4 mV drive amplitude and 290 kHz nominal resonance frequency were used. AFM samples were prepared by dropping 10 μL of MON suspension onto a freshly cleaved mica substrate surface heated at 10 K above the boiling point of solvent used.

### Fourier Transform Infrared

Fourier transform infrared (FT-IR) spectra were recorded on a PerkinElmer Spectrum 100 FT-IR spectrophotometer fitted with a Sensei diamond ATR module.

### GUF-14 (**hxl**) General Synthetic Procedure

ZrCl_4_ (26 mg, 0.1125 mmol) and EDB-H_2_ (30 mg, 0.1125 mmol) were suspended in 4 mL of DMF in a screw-capped glass vial and sonicated for 30 s. The mixture was then transferred to a Teflon lined stainless-steel autoclave with additional 1 mL DMF for washing (5 mL total volume DMF). After addition of deionized water (0.5–2.5% *v*/*v*) and neat formic acid (105–130 equiv) the autoclave was heated overnight at 373 K for 17 h in a preheated oven. After completion of the reaction, the product obtained was isolated by centrifugation (4500 rpm, 10 min), followed with sequential washes: 2× DMF (10 mL each) and 3× acetone (10 mL each) and dried under vacuum inside a desiccator. Optimization of the synthesis is detailed in the Section S3.

### GUF-14 (**hns**) Delamination

Bulk GUF-14 (**hxl**) was activated at 473 K in a programmable oven for 16 h prior to exfoliation to aid transition toward GUF-14 **hns**. Ultrasound-assisted liquid-phase exfoliations were carried out by suspension of 3–3.5 mg of GUF-14 **hxl** in 8 mL of H_2_O or EtOH inside a 10 mL reaction vial. The samples were mixed in a vortex mixer for 30 s to disperse the MOF. The samples were sonicated using a Fisherbrand Elmasonic P 30H ultrasonic bath (2.75 L, 320 W) filled with water. Samples were sonicated for 12 h at a frequency of 37 kHz with 100% power, and the temperature was thermostatically maintained at 289–293 K using a steel cooling coil. Sonication was applied using a sweep mode, and samples were rotated through the water using an overhead stirrer. Suspensions of nanosheets were obtained by cascade centrifugation at 1500 rpm for 1 h and 4500 rpm for 2 h, followed by removal of the suspension from the isolated bulk powder.

### Catalysis

Acid washing of GUF-14 (**hxl** and **hns** samples) was performed by following the procedure reported by Stoddart and co-workers.^57^ All catalysts were activated at 423 K under a vacuum prior to catalysis.

### Imine Catalysis

In a Schlenk tube equipped with a magnetic stir bar, either 0.5 mol % GUF-14 **hns** MONs, 1 mol % Zr-EDB **fcu**, or 0.5 mol % GUF-14 **hxl** were added to 3 mL of dry, degassed toluene and dispersed via sonication for 2 h. One mmol of 4′-fluoroacetophenone, 1 mmol of benzylamine and 0.5 mmol of 1-methylnaphthalene were added to the mixture and heated at 363 K for 16 h under a nitrogen atmosphere with continuous stirring. The conversion was determined by ^19^F{^1^H} NMR spectroscopy.

### DMNP Hydrolysis

DMNP hydrolysis was carried out by adapting the procedure reported by Farha and co-workers.^60^ GUF-14 **hns** MONs (6 mol %) was added to a mixture of 1.05 mL of *N*-ethyl morpholine solution (0.05 mL) deionized H_2_O (0.9 mL) and D_2_O (0.1 mL) in a 2 mL vial. The resulting mixture was sonicated for 2–3 min to ensure a uniform dispersion of the MONs. DMNP (4.0 μL) was added to this suspension and again sonicated for 20 s. The reaction mixture was then transferred to an NMR tube, and the spectrum was measured as quickly as possible. The reaction progress was monitored by ^31^P{^1^H} NMR spectroscopy.

## Supporting Information Available

Crystal structure, synthesis optimization, DFT analyses, exfoliation, catalysis (PDF)
(CIF)

## Supplementary Material

am4c00604_si_001.pdf

am4c00604_si_002.cif
