# Supplementary material for: Modulated Self-Assembly of Catalytically Active Metal–Organic Nanosheets Containing Zr6 Clusters and Dicarboxylate Ligands
Source: ACS Appl Mater Interfaces. 2024 Apr 1;16(14):17812–20. doi: 10.1021/acsami.4c00604 (PMC11009912; doi:10.1021/acsami.4c00604)
Supplement: Supplementary file 1 — am4c00604_si_001.pdf [file am4c00604_si_001.pdf]

SUPPORTING INFORMATION

Modulated Self-Assembly of Catalytically Active Metal-Organic Nanosheets Containing Zr_6 Clusters and Dicarboxylate Ligands

Ram R. R. Prasad,[a] Sophia S. Boyadjieva,[b] Guojun Zhou,[d] Jiangtian Tan,[a] Francesca C. N. Firth,[e] Sanliang Ling,[f] Zhehao Huang,[d] Matthew J. Cliffe,[c] Jonathan A. Foster[a*] and Ross S. Forgan[b*]

[a]Department of Chemistry, The University of Sheffield, Sheffield, S3 7HF, UK. Email: jona.foster@sheffield.ac.uk

[b]WestCHEM School of Chemistry, University of Glasgow, Joseph Black Building, University Avenue, Glasgow, G12 8QQ, UK. Email: ross.forgan@glasgow.ac.uk

[c]School of Chemistry, University of Nottingham, University Park, Nottingham, NG7 2RD, UK.

[d]Department of Materials and Environmental Chemistry, Stockholm University, Stockholm, SE-10691 Sweden.

[e]Yusuf Hamied Department of Chemistry, University of Cambridge, Cambridge CB2 1EW, UK.

[f]Advanced Materials Research Group, Faculty of Engineering, University of Nottingham, University Park, Nottingham NG7 2RD, UK.

Table of Contents

S1. Synthesis. **S2.**

S1.1. Linker Synthesis. **S2.**

S1.2. Synthesis of fcu Topology Zr-EDB. **S4.**

S2. Structural Identification. **S5.**

S3. Synthetic Optimisation. **S8.**

S3.1. Synthetic Variables **S8.**

S3.2. DFT Calculations **S9.**

S3.3. Bulk Characterisation **S11.**

S4. Synthetic Attempts with Alternative Linkers and Modulators. **S18.**

S5. Ultrasound-Assisted Liquid Phase Exfoliation. **S21.**

S6. Catalysis. **S25.**

S6.1. Acid Wash Procedure. **S25.**

S6.2. Imine Catalysis. **S27.**

S6.3. DMNP Hydrolysis. **S40.**

S7. References. **S45.**

S1. Synthesis

S1.1. Linker Synthesis

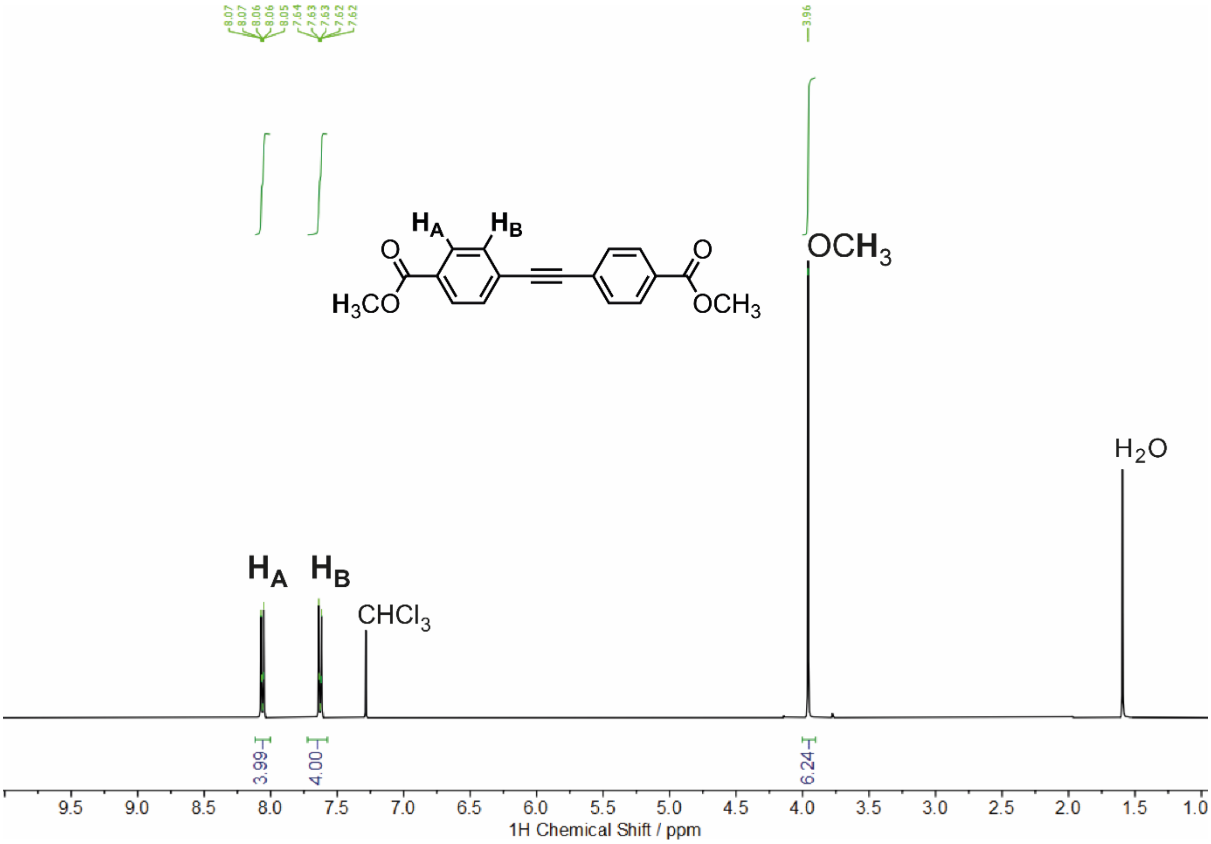

Figure S1. ^{1}H NMR spectrum (CDCl$_3$, 298 K) of dimethyl 4,4′-(ethyne-1,2,-diyl)dibenzoate.

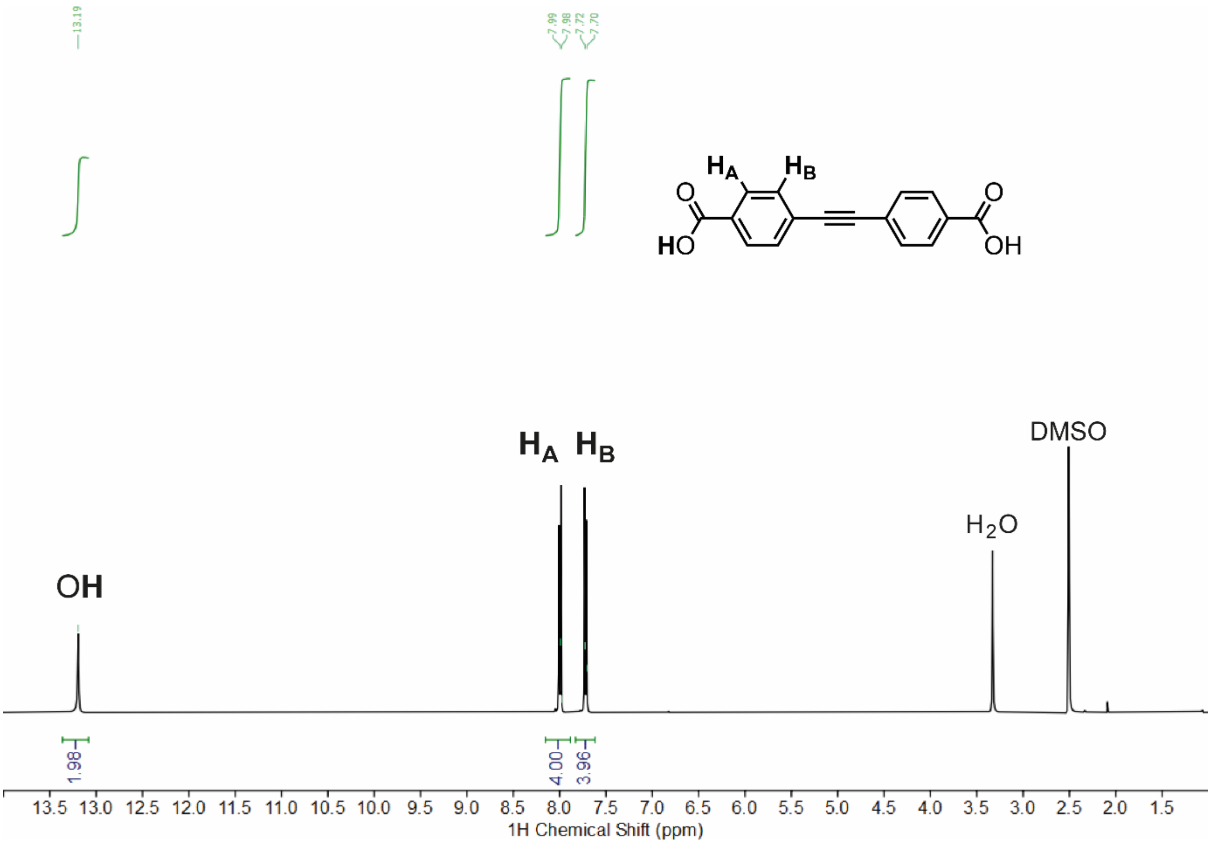

Figure S2. ^{1}H NMR spectrum (DMSO-d_6, 298 K) of 4,4′-(ethyne-1,2,-diyl)dibenzoic acid (EDB-H_2).

S1.2. Synthesis of fcu Topology Zr-EDB

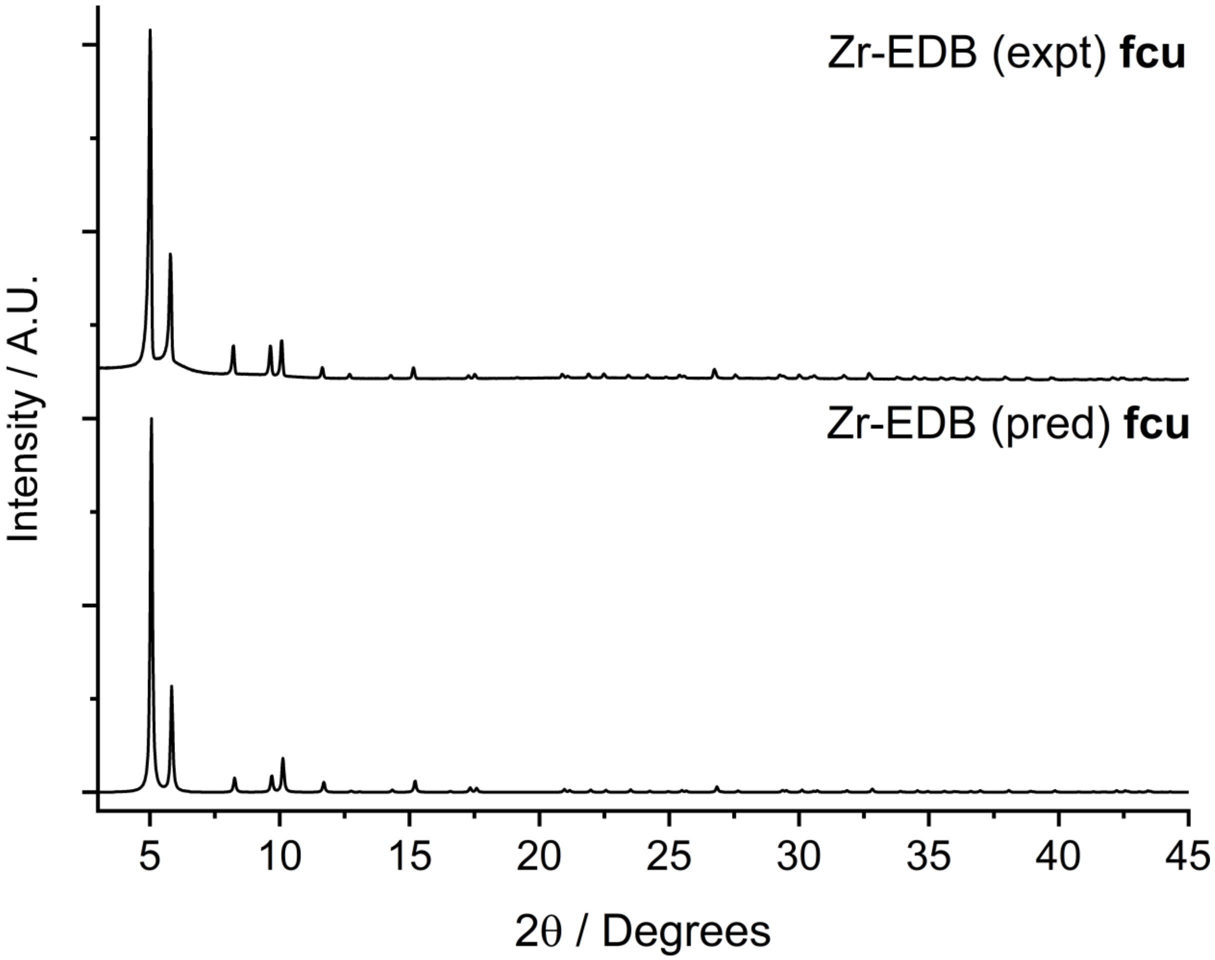

Figure S3. Powder X-ray diffractogram for the synthesised Zr-EDB **fcu** phase sample compared to the pattern predicted from its single crystal structure.[S1]

S2. Structural Identification

Phase identification was possible by continuous rotation electron diffraction (cRED) analysis of a sample of GUF-14 prepared using 125 equiv of formic acid modulator and with addition of 0.5% *v*/*v* deionised water.

Due to the intergrowth of the particles, a fragment was used for structural analysis. Figure S4 shows the reconstructed 3D reciprocal lattice from the cRED data that GUF-14 has a primitive unit cell with the parameters of a = 21.11 Å, b = 21.10 Å, c = 14.74 Å, α = 89.88°, β = 89.78°, and γ = 120.98°. As the lattice parameters a and b are very similar, with α and β close to 90°, and γ close to 120°, it indicates that the possible crystal system could be trigonal or hexagonal.

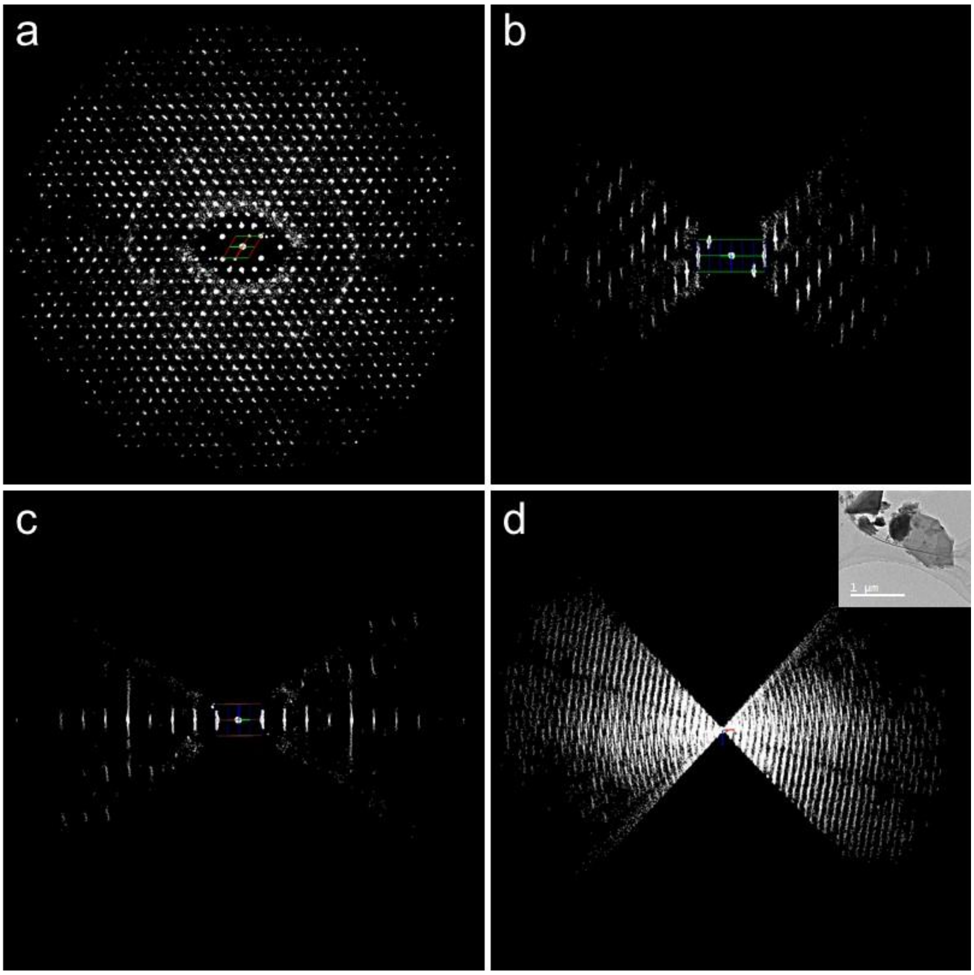

Figure S4. 2D slice cuts from the reconstructed 3D reciprocal lattice of GUF-14 show the (a) *hki*1, (b) *h*-*h*0*l*, and (c) *hh*-2*hl* planes. (d) Projection of the reconstructed 3D reciprocal lattice. Inset is image of the crystal from which the cRED data was collected.

The two-dimensional (2D) slice cuts of the 3D reciprocal lattice at *hki*1, *h*-*h*0*l*, and *hh*-2*hl* planes show reflection conditions $-h + k + l = 3n$, $h + l = 3n$, $l = 3n$, respectively, which confirms the trigonal unit cell (Figures S4a, S4b and S4c). Thus, there are several possible space groups for GUF-14: $R3$ (No. 146), $R\bar{3}$ (No. 148), $R32$ (No. 155), $R3m$ (No. 160), and $R\bar{3}m$ (No. 166). The space group $R\bar{3}m$ with highest symmetry was chosen for further structural determination.

The ED data were processed using the XDS package. The dataset has a high signal-to-noise ratio within the resolution of 0.90 Å. Due to the preferred orientation of the 2D crystal, and the limitation of goniometer tilting range (Figure S4d), the data completeness is 85.6%. The cRED data has sufficient quality to determine the framework structure of GUF-14 by direct methods using the program Shelx-2017. The final refinement was done by using Shelxl-2017, and data converged to $R_1 = 0.284$. The rather high R_1 value is mainly caused by the dynamical effects from the electron beam. In the refinement, the structure factor was calculated in a kinematic approximation, while the electron diffraction data is dynamic.[S2] The details of data collection and refinement are summarized in Table S1. The structure has been deposited with the CSD, deposition number 2314111.

Table S1.

Chemical Formula	$C_{16.62}H_{11.33}O_{10.67}Zr_2$
Formula weight	564.19
Crystal system	Trigonal
Space Group	$R\bar{3}m$
a (Å)	21.007(3)
b (Å)	21.007(3)
c (Å)	14.647(3)
α (°)	90
β (°)	90
γ (°)	120
Volume (Å^3)	5597.7(19)
Z	9
Wavelength (Å)	0.0251
Radiation type	electron
Temperature (K)	293(2)
Completeness (%)	85.6
No. of reflections (all unique)	903
No. of reflections (Fo>4σ(Fo))	9402
Refined parameters	47
R_{int}	0.292
R_1 (Fo>2σ(Fo))	0.3256
R_1 (all reflections)	0.4045
GooF	2.716

S3. Synthetic Optimisation

S3.1. Synthetic Variables

Varying the quantities of formic acid added to solvothermal syntheses did not significantly affect product formation (Figure S5), but increased water content led to lower quality samples of the GUF-12(Zr) **hcp** topology material (Figure S6).

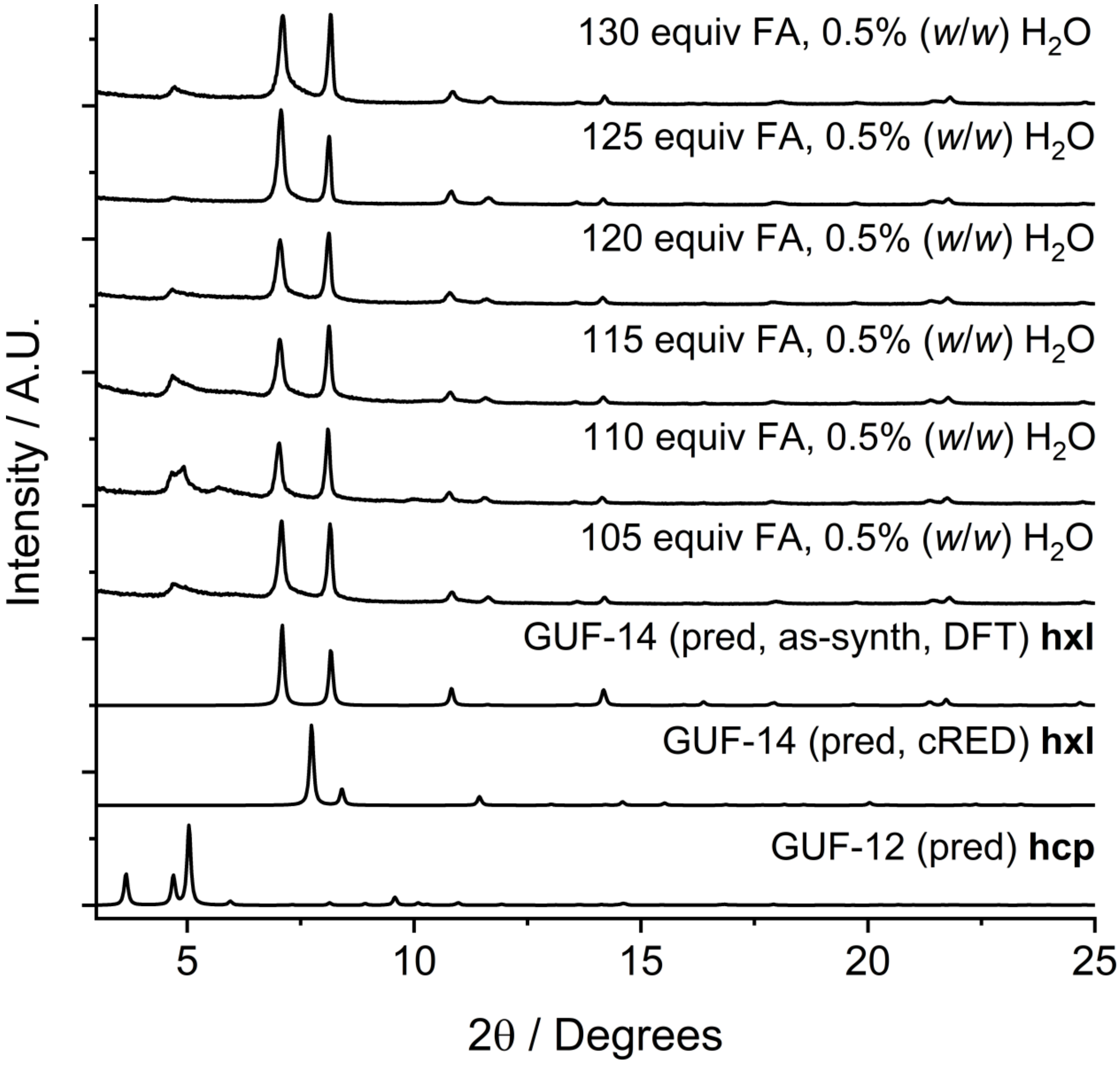

Figure S5. Stacked partial powder X-ray diffractograms of samples of GUF-14 prepared via the general synthetic procedure with addition of 0.5% (*v*/*v*) water and varying amounts of formic acid as modulator, compared to predicted patterns for GUF-14 (cRED structure), as-synthesised GUF-14 (DFT model) and GUF-12 (DFT model).[S3]

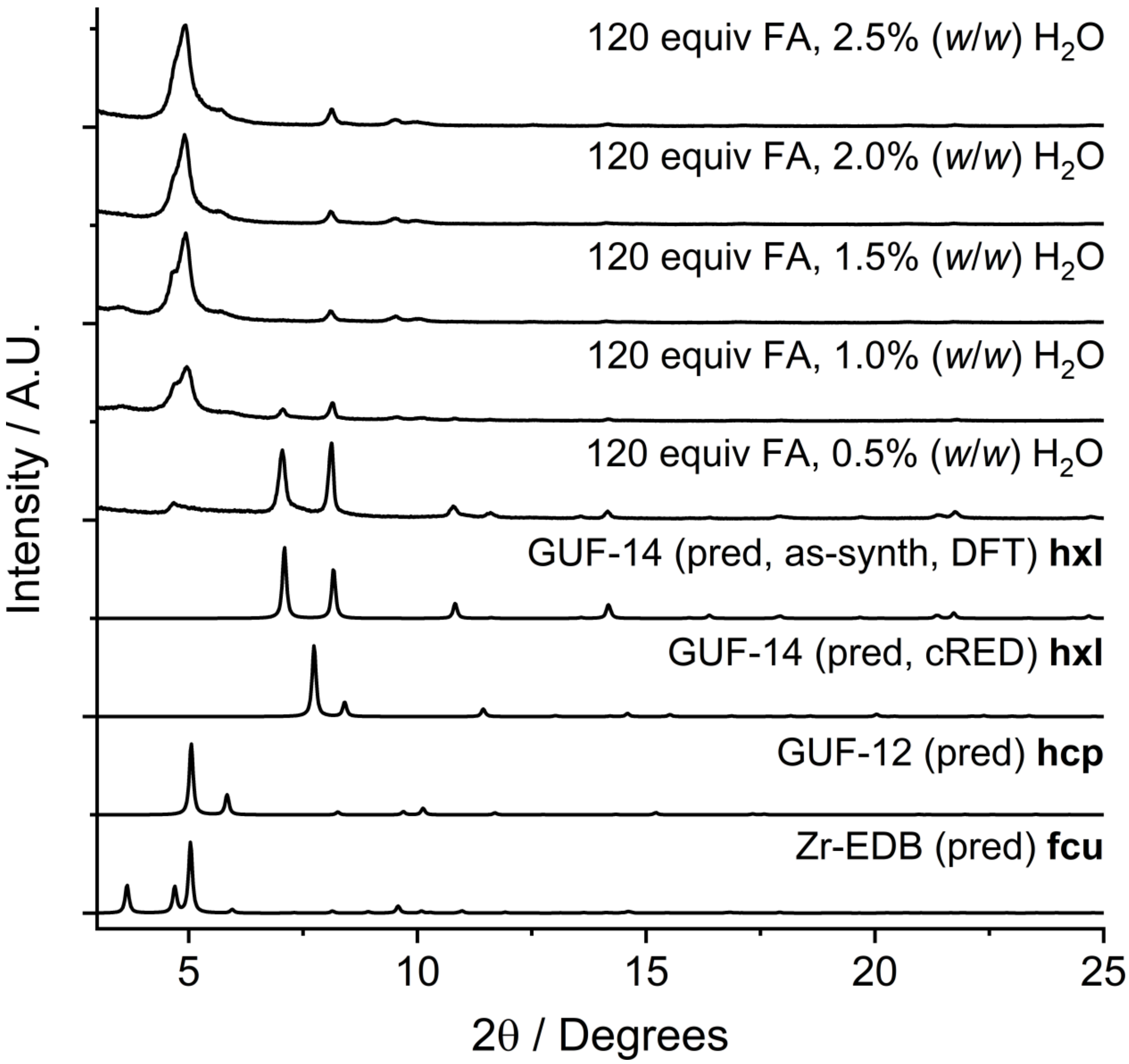

Figure S6. Stacked partial powder X-ray diffractograms of samples of GUF-14 prepared via the general synthetic procedure with addition of 120 equiv formic acid (FA) and varying amounts water, compared to predicted patterns for GUF-14 (cRED structure), as-synthesised GUF-14 (DFT model) GUF-12 (DFT model),[S3] and Zr-EDB **fcu** phase (single crystal structure, X-ray diffraction).[S1]

S3.2. DFT Calculations

All density functional theory (DFT) calculations have been performed using the CP2K code, which uses a mixed Gaussian/plane-wave basis set.[S4, S5] We employed double-

ζ polarization quality Gaussian basis sets[S6] and a 400 Ry plane-wave cutoff for the auxiliary grid, in conjunction with the Goedecker-Teter-Hutter pseudopotentials.[S7, S8] All DFT calculations were performed in the Γ-point approximation with a sufficiently large supercell. Total energy calculations and structural optimizations, including both atomic coordinates and cell parameters, were performed under periodic boundary conditions at the DFT level using the PBE exchange and correlation functional,[S9] with Grimme's D3 van der Waals correction (PBE+D3).[S10] A convergence threshold of 1.0 $\times 10^{-6}$ Hartree was used for the self-consistent field cycle, and structural optimizations were considered to have converged when the maximum force on all atoms falls below 4.5×10^{-4} Hartree/Bohr.

We considered three different types of capping units on the Zr_6 SBUs of GUF-14, including formate, hydroxide, and hydroxide with guest water, which were generated by modifying an existing model of a Zr_{12} cluster containing **hxl** topology MOF while maintaining overall charge balance. Our DFT calculations show that when all the three cell parameters along *a*, *b* and *c* axes are allowed to relax, the interlayer spacing of GUF-14, which determines the lattice parameter along the *c* axis, is very sensitive to the identity of the capping unit, being either formate, hydroxide, or hydroxide with water. To assess the phase purity of the as-synthesised GUF-14 material, we performed an additional cell optimisation based upon the fully optimised DFT structure of GUF-14 with formate as the capping unit, but we fixed the *c* axis parameter to 16.66 Å, derived from the Pawley fit in Figure 3a, during the partial cell optimisation while allowing the cell parameters along *a* and *b* axes to relax. We refer this structure as the DFT model of the as-synthesised GUF-14.

A comparison of the diffractograms for the DFT model of the as-synthesised GUF-14 and the experimental data for the sample prepared with 125 equivalents of FA and 0.5% (*w/w*) water is given in Figure S7.

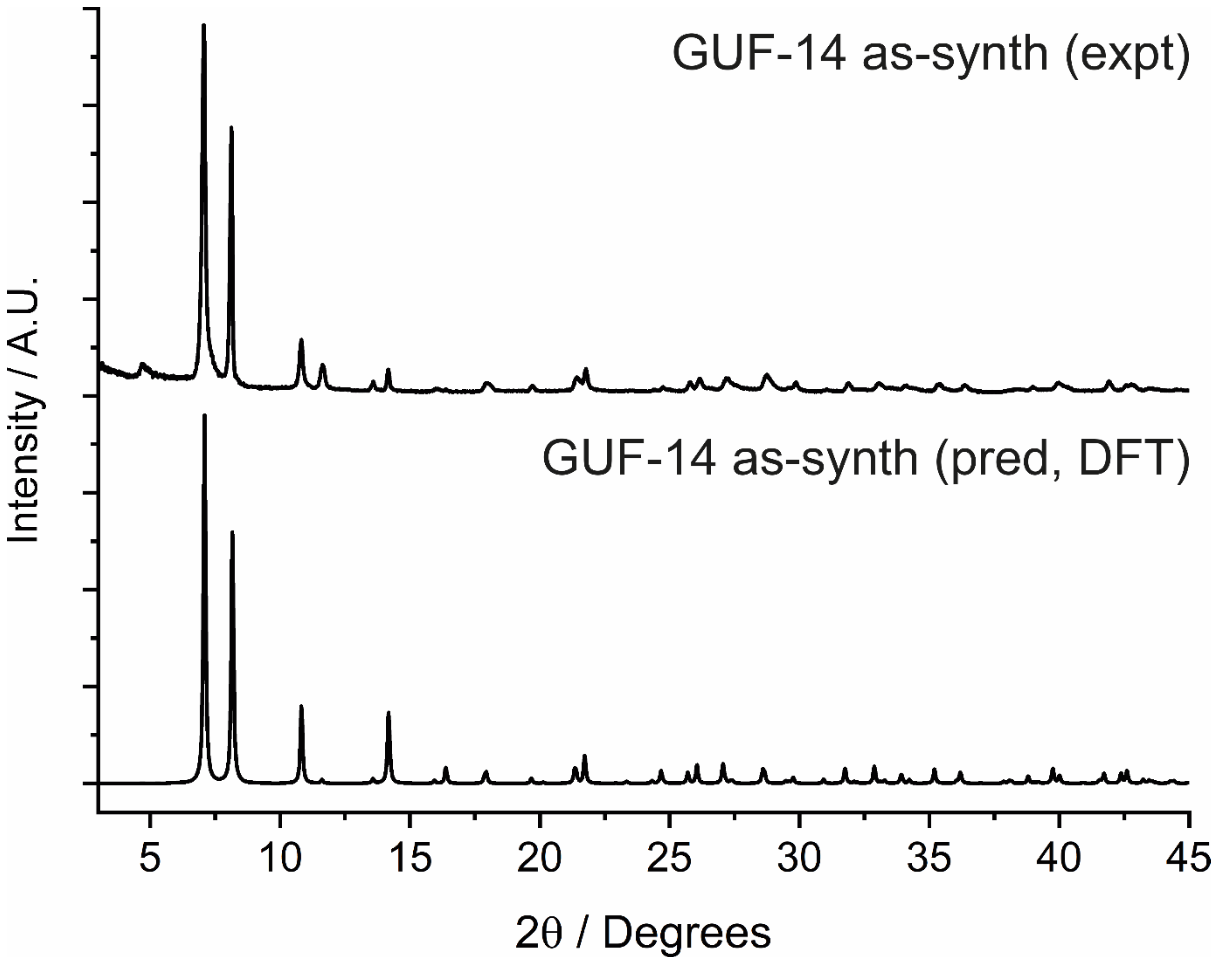

Figure S7. Comparison of the powder X-ray diffractograms for as-synthesised GUF-14 predicted from the DFT model (with an applied March-Dollase parameter of 1.5 along the 001 direction to mimic preferred orientation in the hexagonal plate material) with the experimental data for a sample of GUF-14 prepared via the general synthetic procedure with addition of 0.5% (*v*/*v*) water and 125 equiv of formic acid as modulator.

S3.3. Bulk Characterisation

^{1}H NMR spectroscopy of acid-digested samples (Figure S8) was used to confirm the integrity of the EDB^{2-} linker and the incorporation of formate, which increased with the content of formic acid in synthesis.

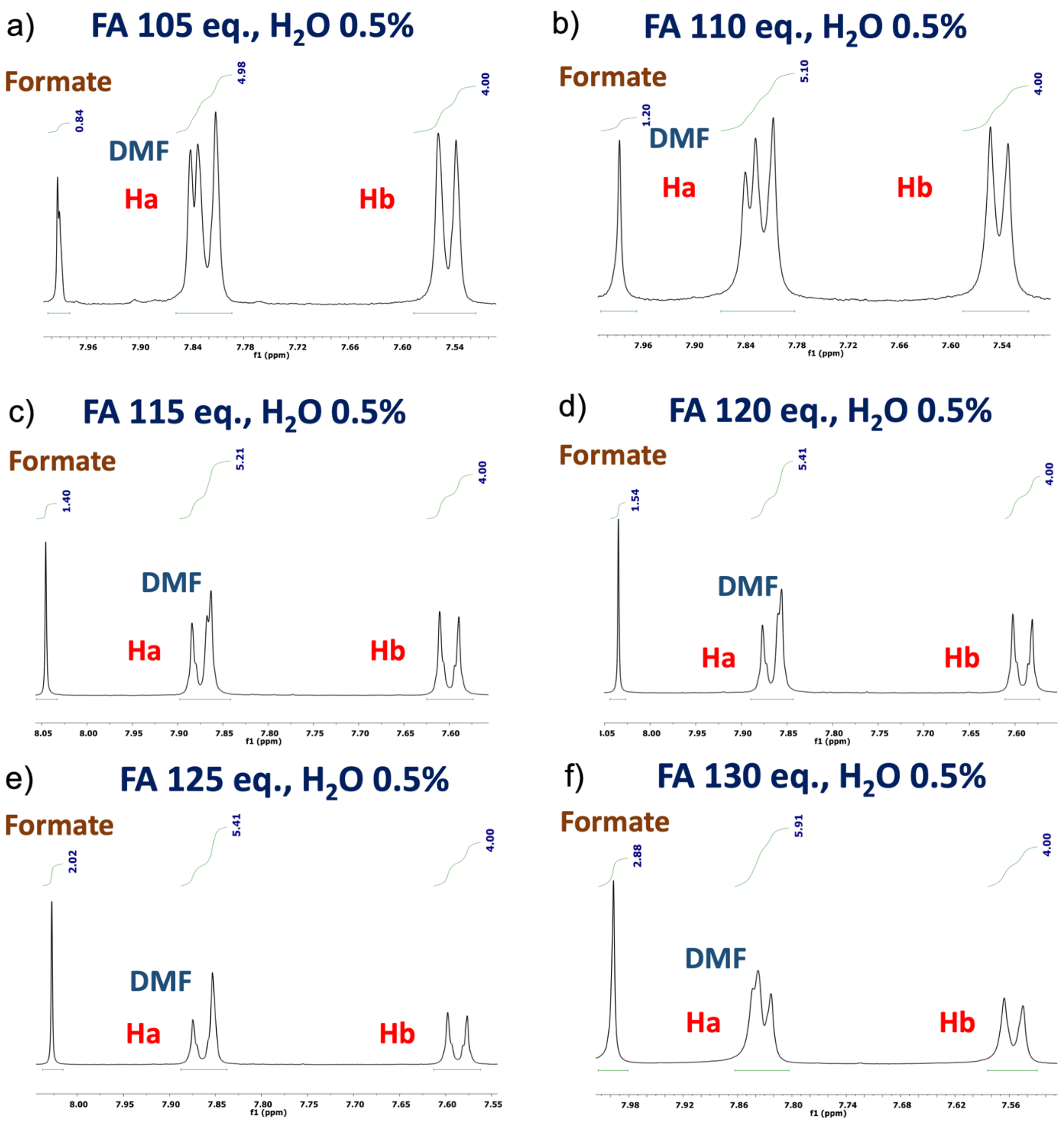

Figure S8. Partial ^{1}H NMR spectra (400 MHz, 298 K) of acid digested (DMSO-d_6 / D_2SO_4) samples of GUF-14 prepared via the general synthetic procedure with addition of 0.5% (*v*/*v*) water and varying amounts of formic acid as modulator. Integrals for the two non-equivalent aromatic protons of the EDB-H_2 linker (Ha and Hb) show increased formic acid content in as-synthesised samples as the formic acid content in syntheses increases. A "fully capped" Zr_6 SBU would yield a MOF with ideal formula $[Zr_6(\mu_3\text{-}O)_4(\mu_3\text{-}OH)_4(HCO_2)_6(EDB)_3]$ and an EDB^{2-}: $HCOO^-$ ratio of 1:2. Ratios in the spectra are a) 1:0.8, b) 1:1.2, c) 1:1.4, d) 1:1.5, e) 1:2.0, and f) 1:2.9. The formic acid can both cap the cluster and be present as a guest within the pore structure.

Scanning electron microscopy (SEM) was used to assess the effect of formic acid modulation on particle size and morphology (Figures S9 and S10).

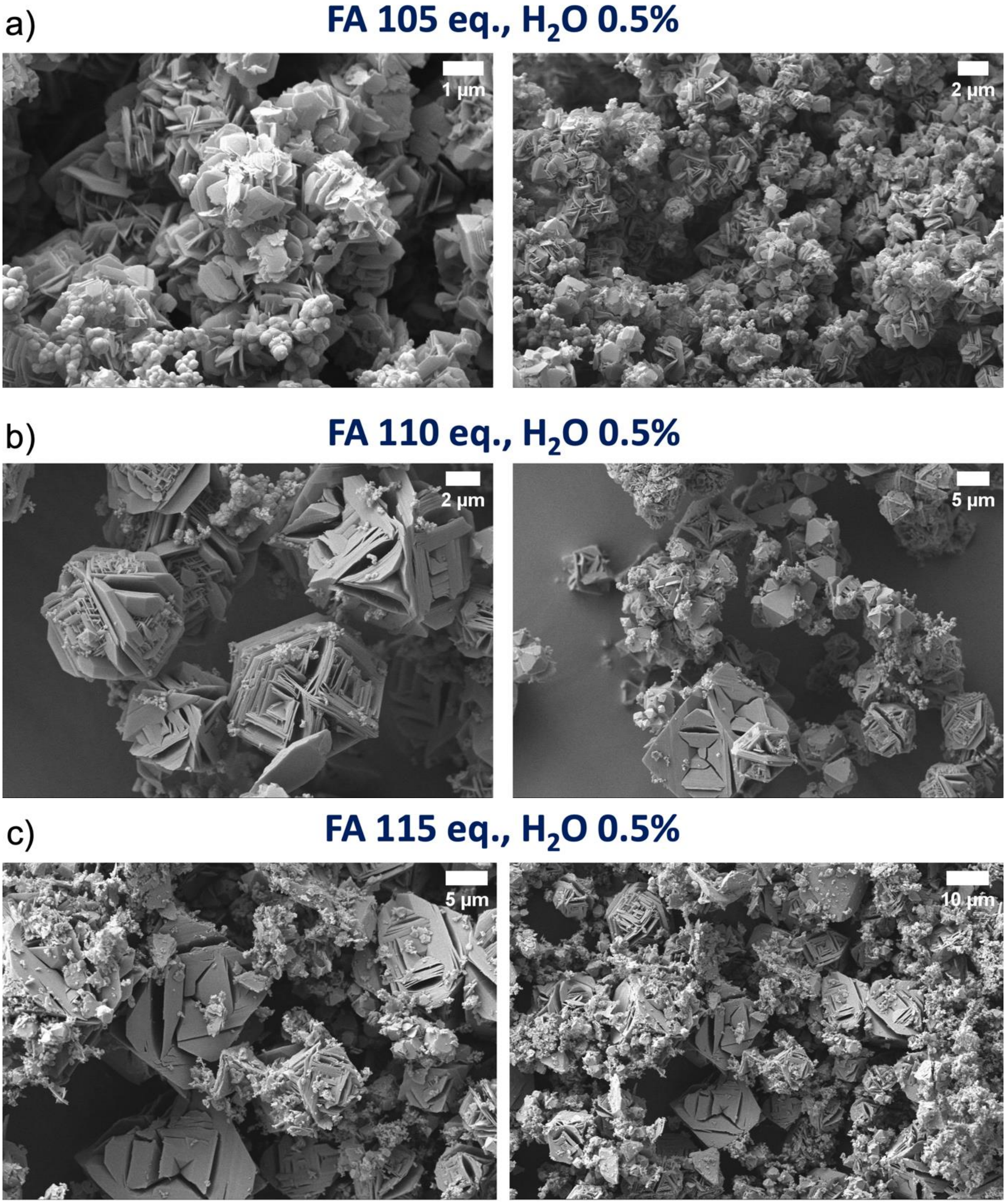

Figure S9. Scanning electron micrographs of samples of GUF-14 prepared using a) 105 equiv formic acid (FA), b) 110 equiv formic acid, and c) 115 equiv formic acid, alongside 0.5% (*v*/*v*) water. Samples correspond to those in Figures S5 and S8. Scale bars as shown.

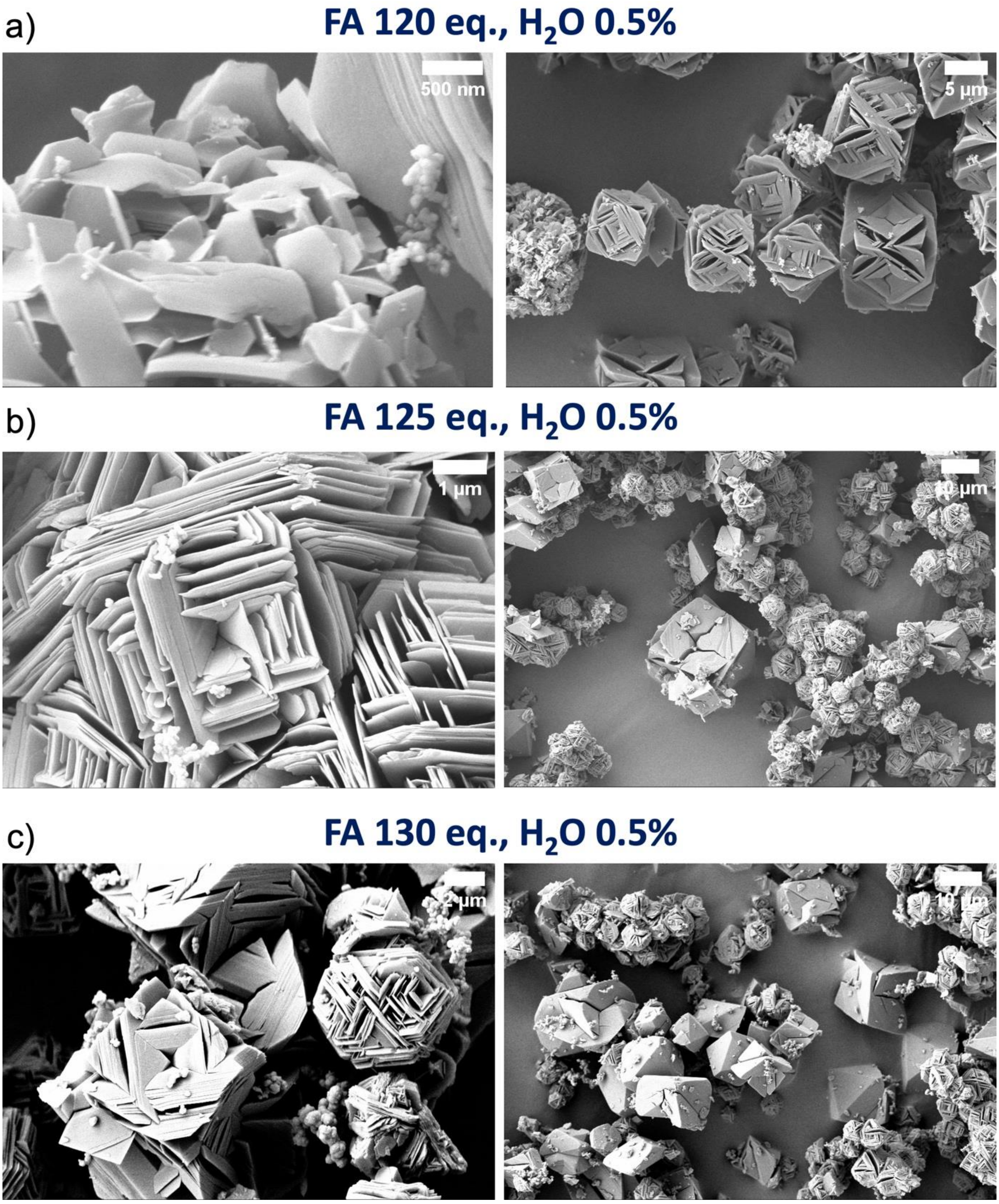

Figure S10. Scanning electron micrographs of samples of GUF-14 prepared using a) 120 equiv formic acid (FA), b) 125 equiv formic acid, and c) 130 equiv formic acid, alongside 0.5% (*v*/*v*) water. Samples correspond to those in Figures S5 and S8. Scale bars as shown.

All samples showed morphology distinct from the well-defined octahedra of the Zr-EDB **fcu** phase and the "desert rose" clusters characteristic of the GUF-12 **hcp** phase.

Agglomerations of very thin plates are present alongside larger particles, suggesting that delamination is already occurring to some extent under the work-up conditions. Very thin sheets are evident, for example, in Figure S10a (left).

The sample prepared with 125 equiv of formic acid and 0.5% (*v*/*v*) water was selected for further characterisation, and all further materials described in this submission used this specific synthesis. Samples were washed with acetone and dried under turbopump vacuum at 393 K for 20 h, before analysis by ^{1}H NMR spectroscopy (Figure S11) and thermogravimetric analysis (Figure S12).

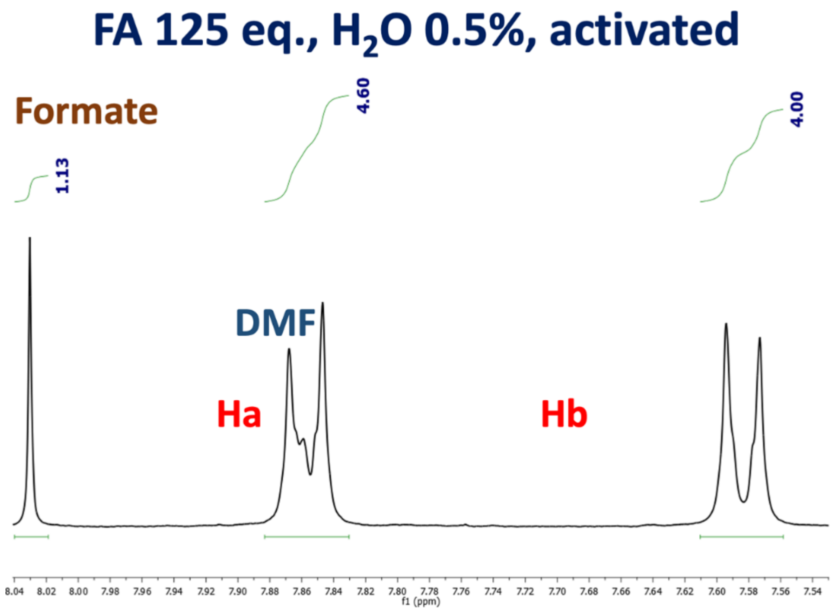

Figure S11. Partial ^{1}H NMR spectrum (400 MHz, 298 K) of an acid digested (DMSO-d_6 / D_2SO_4) sample of GUF-14 prepared via the general synthetic procedure with addition of 0.5% (*v*/*v*) water and 125 equiv of formic acid as modulator. The sample had been activated by heating at 393 K for 20 h under turbopump vacuum prior to analysis. The EDB^{2-}: $HCOO^-$ ratio of 1:1.1 suggests an overall formula for this sample of formula $[Zr_6(\mu_3\text{-}O)_4(\mu_3\text{-}OH)_4(HCO_2)_{3.3}(OH)_{2.7}(OH_2)_{2.7}(EDB)_3]$.

Thermogravimetric analysis in air of the activated GUF-14 sample showed a three-step mass loss process, with the final thermal breakdown occurring around 450 °C (Figure S12). The residual mass of 41.3% wt corresponds well to that predicted from the formula derived from the NMR spectroscopic analysis in Figure S11, which would be 43.1% wt assuming complete conversion to ZrO_2.

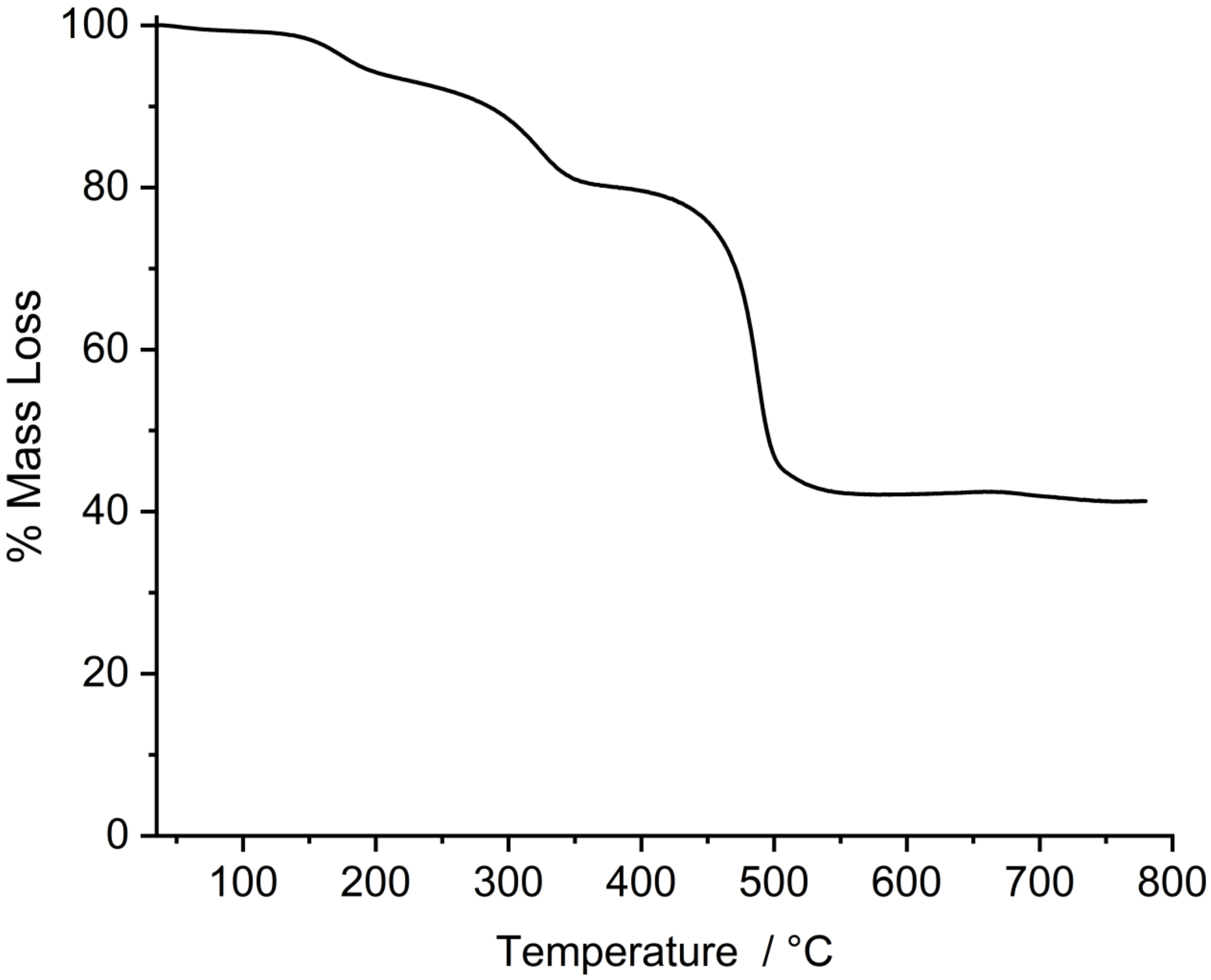

Figure S12. Thermogravimetric analysis in air of a sample of GUF-14 prepared via the general synthetic procedure with addition of 0.5% (*v*/*v*) water and 125 equiv of formic acid as modulator. The sample had been activated by heating at 393 K for 20 h under turbopump vacuum prior to analysis.

Refinement of the cRED structure and DFT-optimised structure in the cells derived from Pawley refinement was carried out using Topas Academic 6.0 (Figure S13). The DFT-derived structure in *P*1 was converted to the experimentally observed rhombohedral $R\bar{3}m$. The atomic positions and thermal parameters were kept fixed for both refinements due to the presence of large number of stacking faults and indeterminate guest composition, which produced systematic intensity variations not modelled in the average structural models (DFT and cRED). The March-Dollase correction along (001) was used due to significant preferred orientation. *hkl* dependent

peak broadening was applied to account for significant peak asymmetry due to stacking faults.

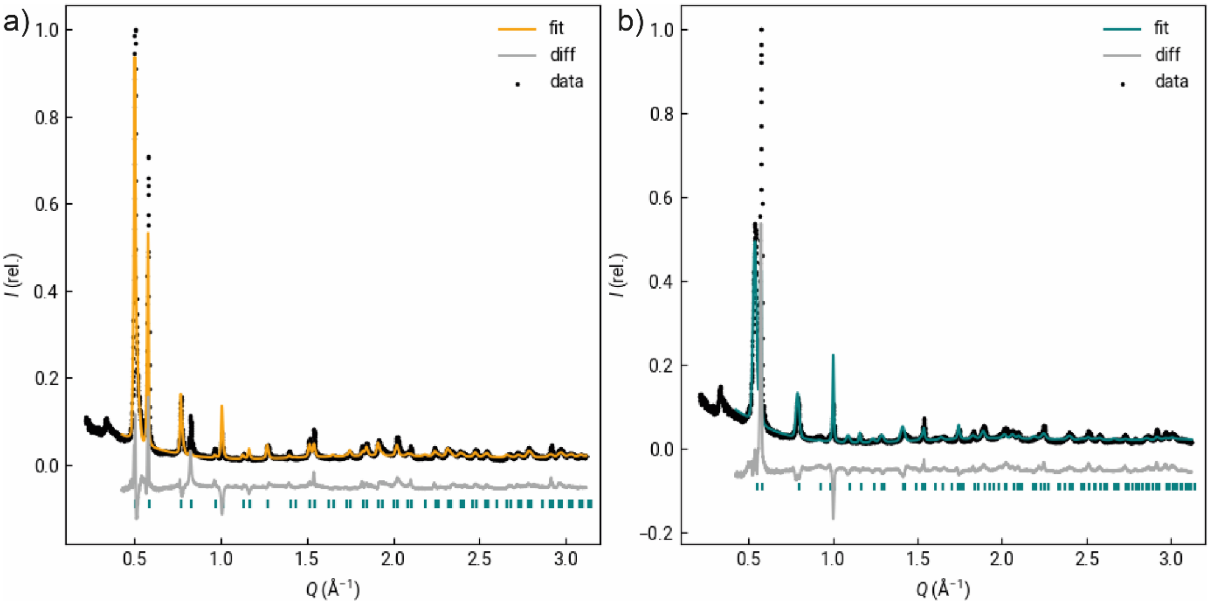

Figure S13. Refinement of PXRD data measured for GUF-14. a) The data for the as-synthesised for refined against the DFT derived model with complete formate occupation and expanded cell. b) Data for an activated sample refined against the cRED model measured in vacuum.

S4. Synthetic Attempts with Alternative Linkers and Modulators

To rationalise the roles of the formic acid modulator and the alkyne spacer of EDB^{2-} in directing the structure of GUF-14, syntheses were attempted under identical conditions to those in the general synthetic procedure, but with different modulators or ligands. The use of acetic acid as modulator is already known to produce GUF-12; here we found that benzoic acid and 3-fluorobenzoic acid, which has a pK_a similar to formic acid, inhibited product formation completely. The use of trifluoroacetic acid (TFA) under the standard reaction conditions led to formation of the Zr-EDB **fcu** phase with a minor, unidentifiable impurity, as indicated by PXRD analysis (Figure S14).

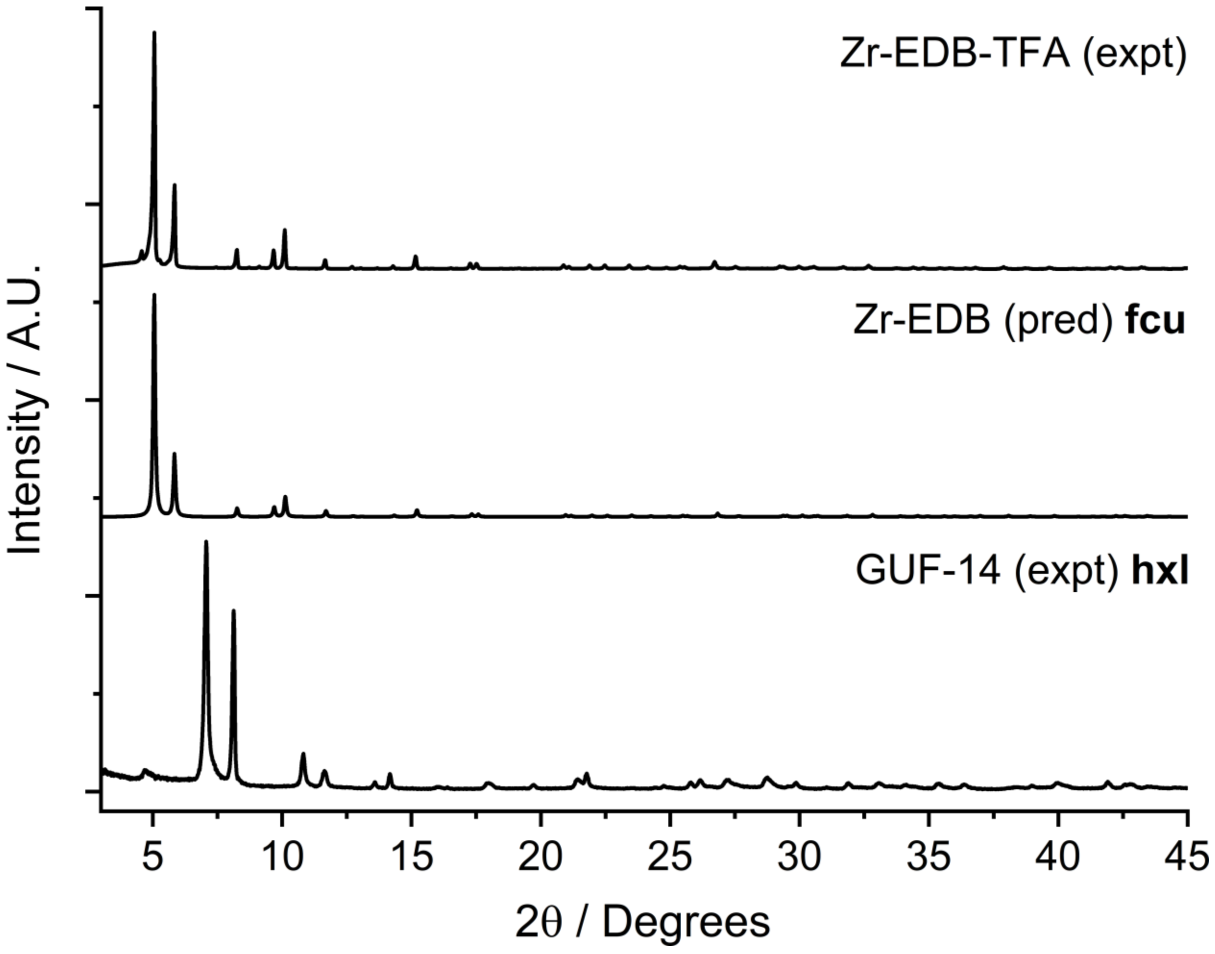

Figure S14. Stacked partial powder X-ray diffractogram of a sample of Zr-EDB synthesised using the general synthetic procedure but with trifluoroacetic acid as modulator, compared to that of an experimental sample of GUF-14 (**hxl**) and a predicted pattern for the Zr-EDB **fcu** phase.[S1]

Two different ligands were used to assess the effect of the alkyne spacer. A formic acid (FA) modulated synthesis using biphenyl-4,4′-dicarboxylic acid (BPDC) led cleanly to the formation of UiO-67, with **fcu** topology (Figure S15), whilst a similar synthesis using acetylenedicarboxylic acid (ADC) gave an amorphous powder (Figure S16). Overall, these experiments further indicate that GUF-14 type phases are only likely to form with the appropriate ligand and modulator combinations.

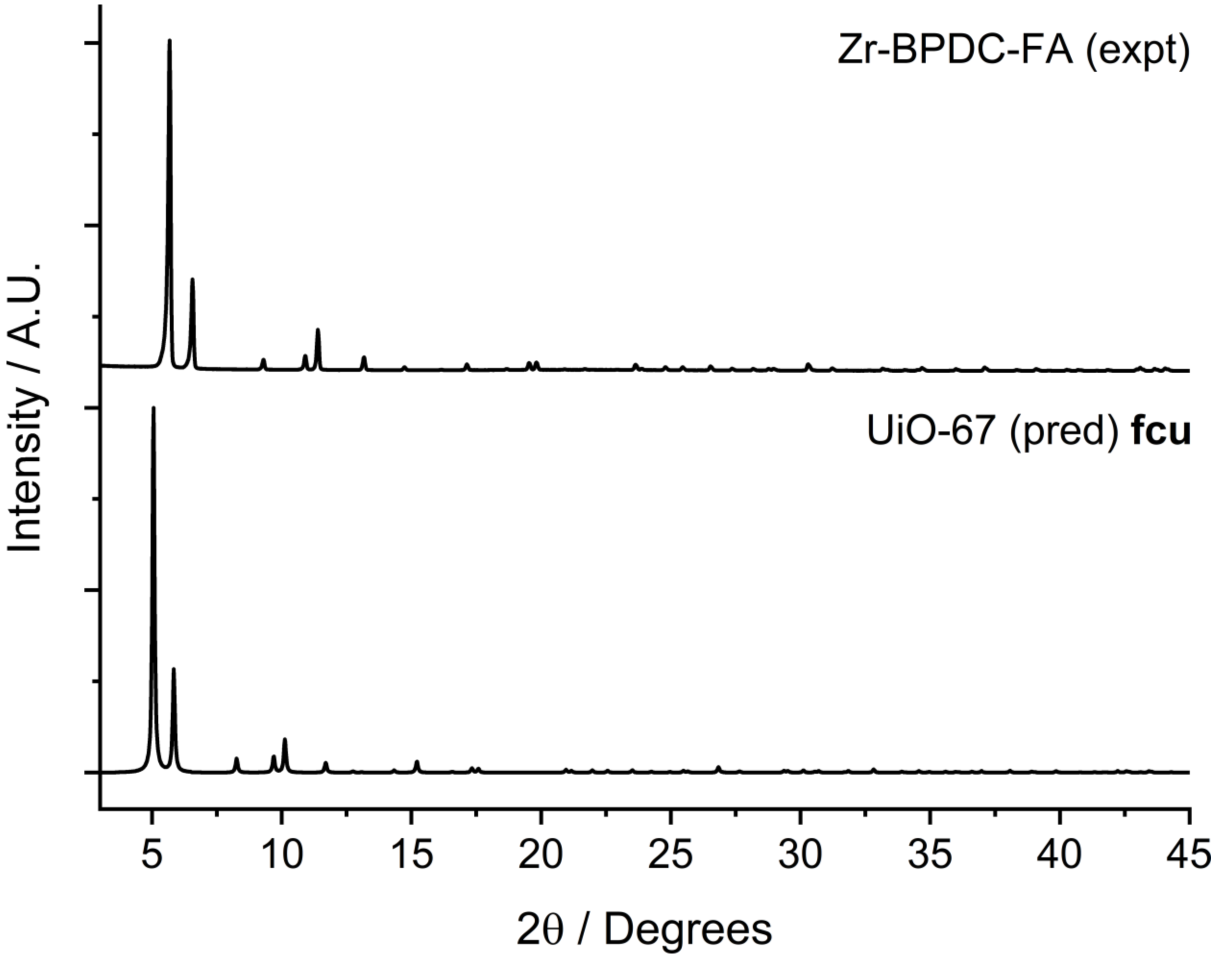

Figure S15. Stacked partial powder X-ray diffractogram of a sample of Zr-BPDC synthesised using the general synthetic procedure with formic acid (FA) as modulator, compared a predicted pattern for **fcu** phase, UiO-67.[S11]

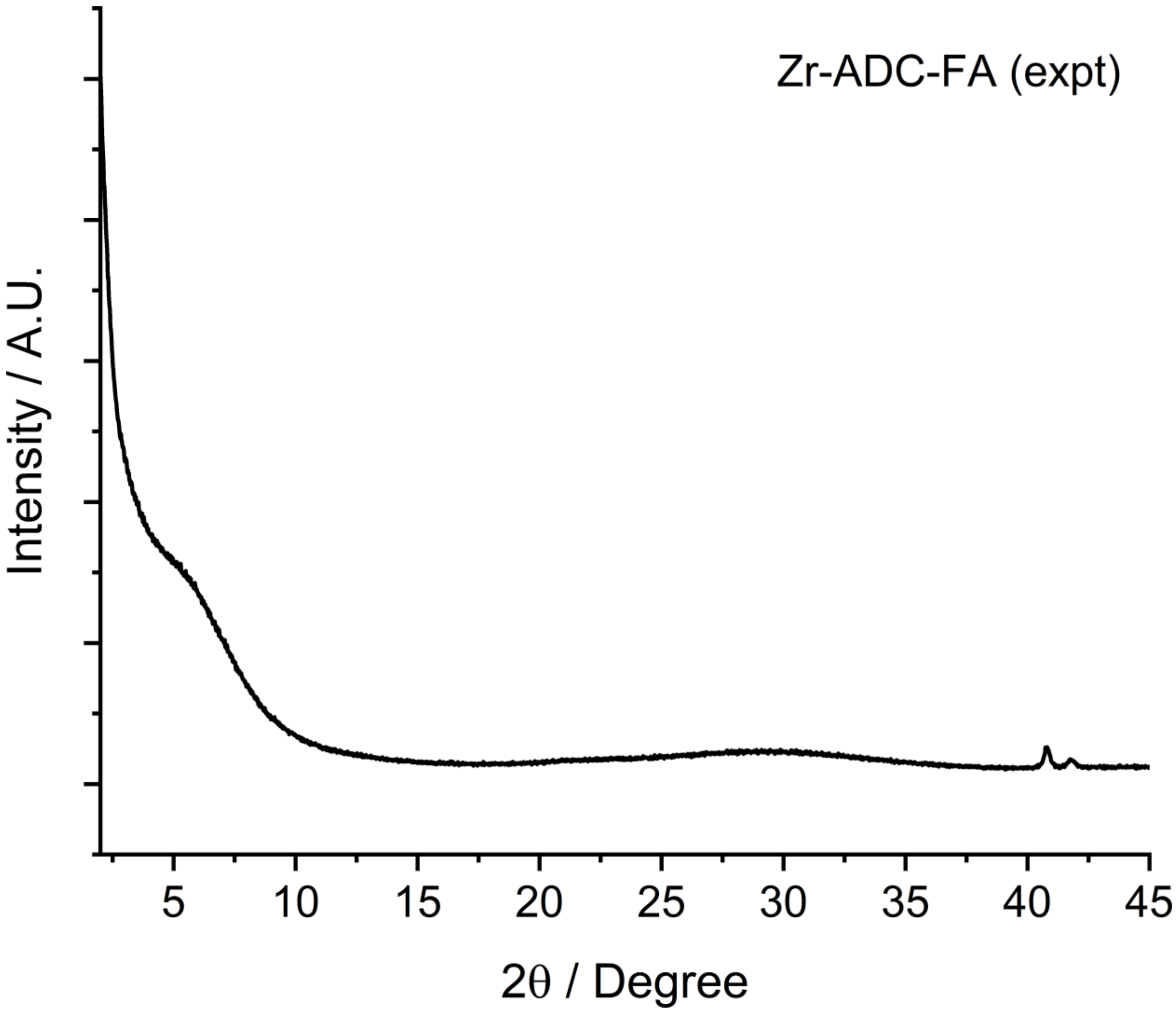

Figure S16. Partial powder X-ray diffractogram of a sample of Zr-ADC synthesised using the general synthetic procedure with formic acid (FA) as modulator. The peaks at 41 and 42° are artifacts arising from the sample holder in the flat plate set-up used.

S5. Ultrasound-Assisted Liquid Phase Exfoliation

AFM samples of GUF-14 (**hns**) nanosheets (Figures S17 and S18) were prepared dropping 10 μL of the suspension onto a freshly cleaved mica substrate surface heated at 10 °C above the boiling point of solvent used.

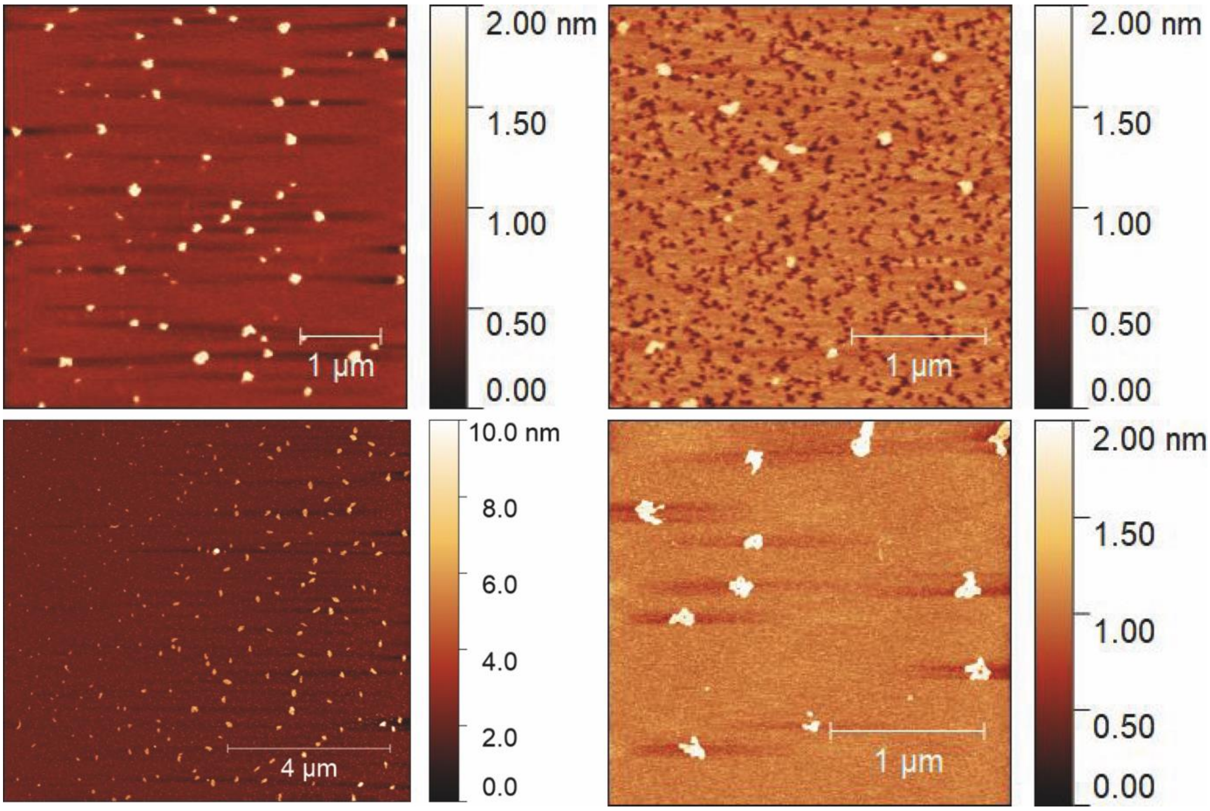

Figure S17. AFM topographic images of the GUF-14 nanosheets (**hns**) exfoliated in water.

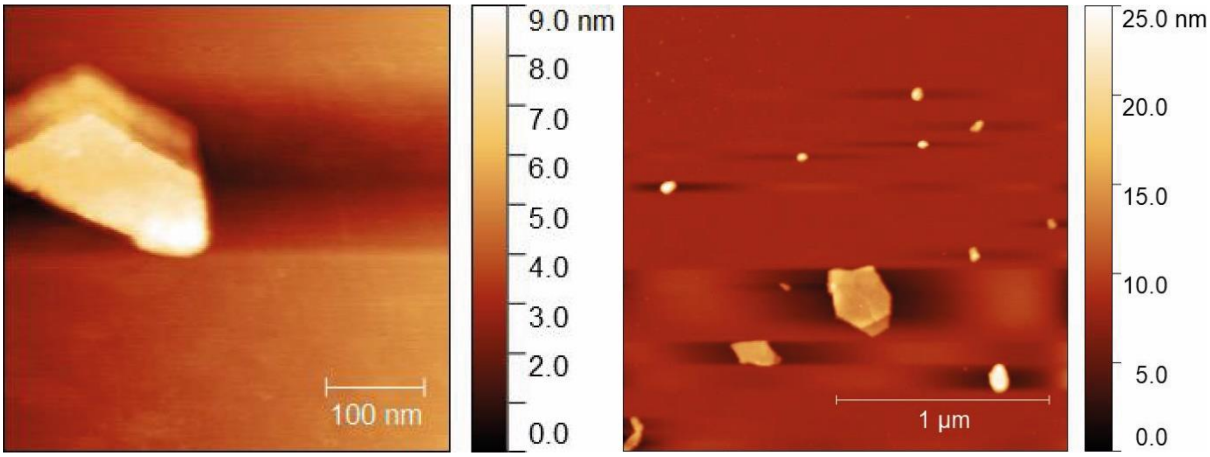

Figure S18. AFM topographic images of the GUF-14 nanosheets (**hns**) exfoliated in ethanol.

The nanosheets exfoliated in water were taken forward for further use, as these preparations led to the thinnest nanosheets (see Figure 4, main manuscript). The integrity of the nanosheets was confirmed by their isolation through centrifugation at 4500 rpm for 2 h and analysis by PXRD (Figure S19) and NMR spectroscopy (Figure S20).

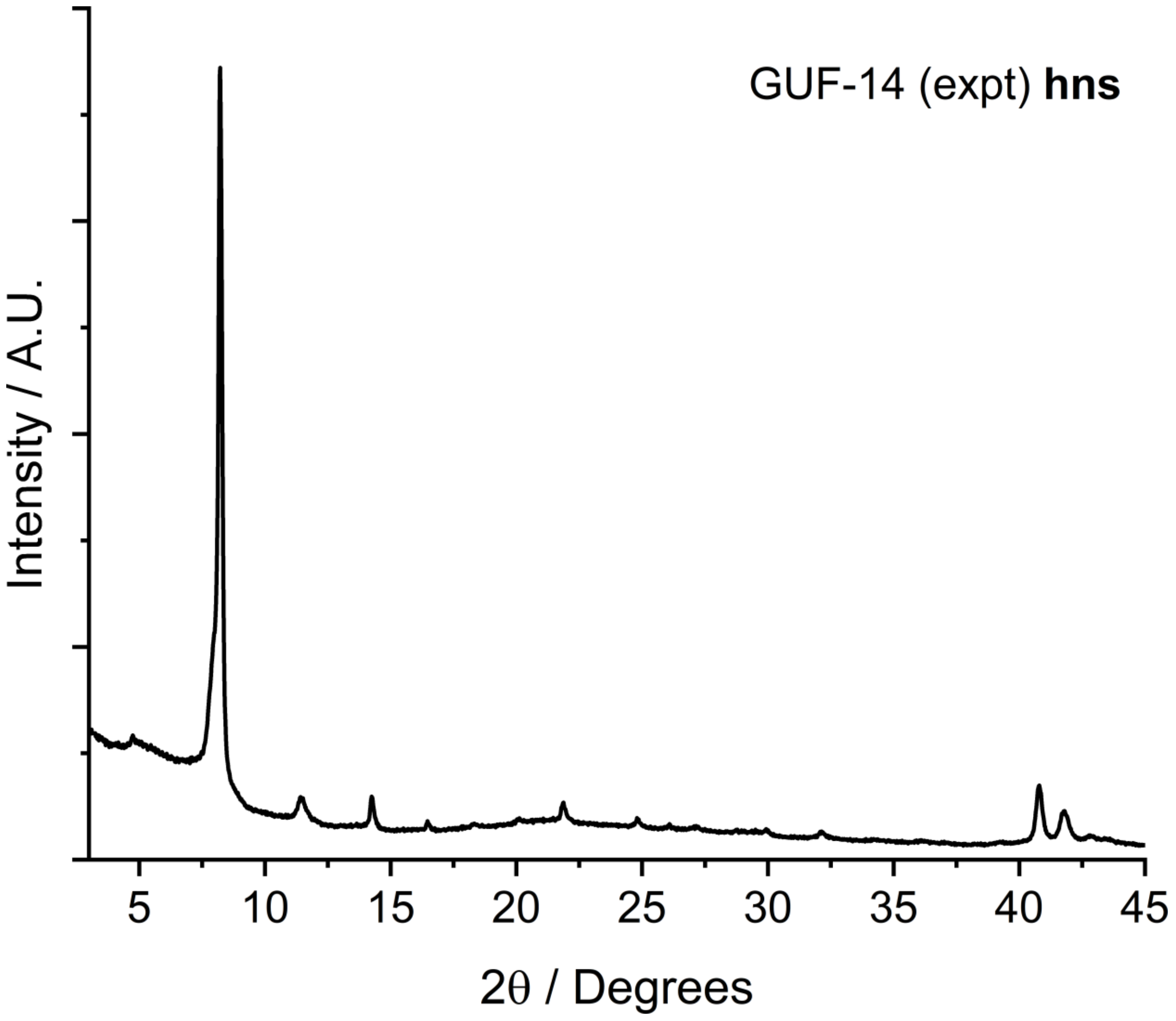

Figure S19. PXRD of GUF-14 hns collected after centrifugation at 4500 rpm for 2 h. The peaks at 41 and 42° are artifacts arising from the sample holder in the flat plate set-up used.

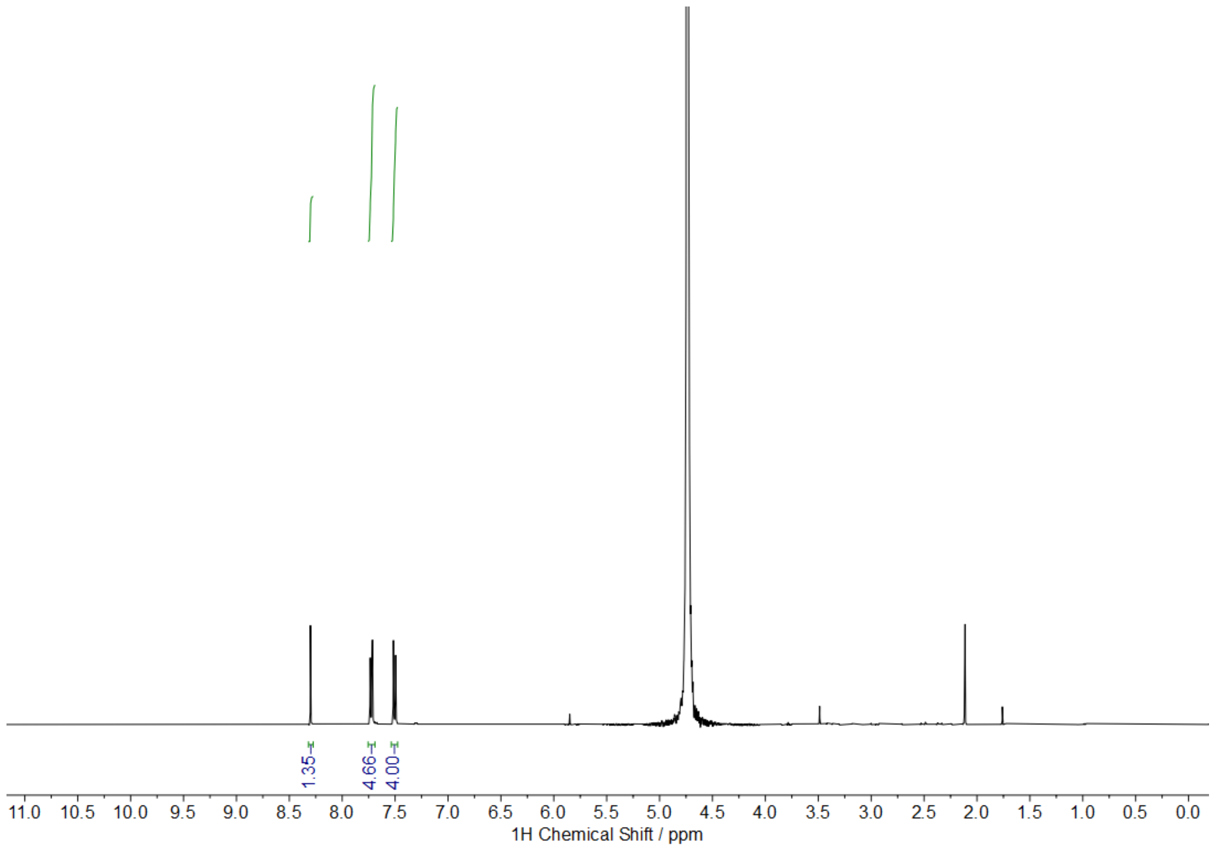

Figure S20. Partial ^{1}H NMR spectrum (400 MHz, 298 K) of a base digested (D_2O / NaOD) sample of GUF-14 nanosheets.

S6. Catalysis

S6.1. Acid Wash Procedure

Acid washing of GUF-14 (**hxl** and **hns** samples) was performed by following the procedure reported by Stoddart and co-workers.[S12] 40 mg of either GUF-14 **hxl** or **hns** was dispersed in 10 mL of 1 M HCl in a screw-capped vial and the vial was heated to 90 °C at a ramp rate of 5 °C/min in a programmable oven. After keeping at 90 °C for 24 h, the vial was taken out of the oven and allowed to cool down to room temperature naturally. The supernatant was removed by centrifugation and the solids washed three times with water to remove any remaining HCl. The samples were kept in acetone for one week with acetone exchanged once a day with fresh batch. After final exchange the samples were dried under vacuum prior to NMR spectroscopic (Figures S21 and S22) and PXRD (Figures S23) analysis.

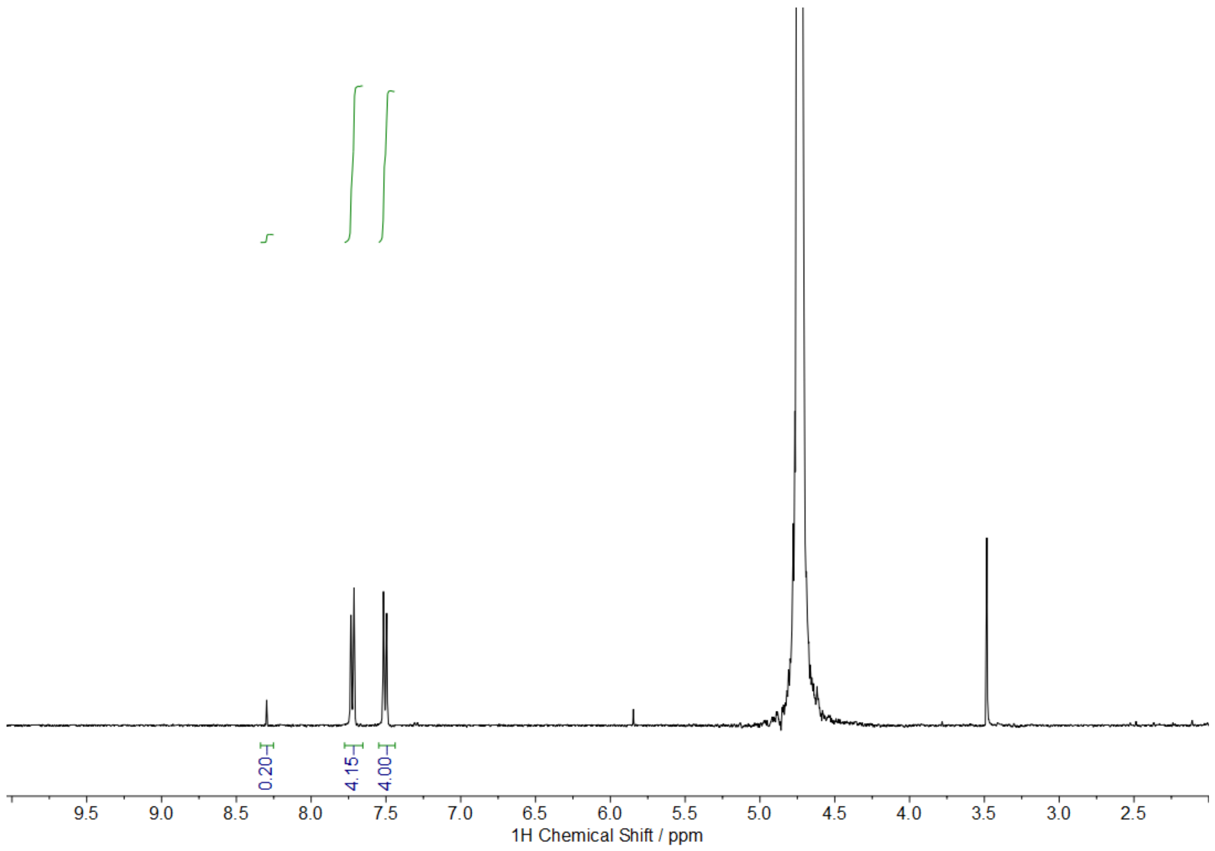

Figure S21. Partial ^{1}H NMR spectrum (400 MHz, 298 K) of a base digested (D_2O / NaOD) sample of GUF-14 **hxl** after acid washing.

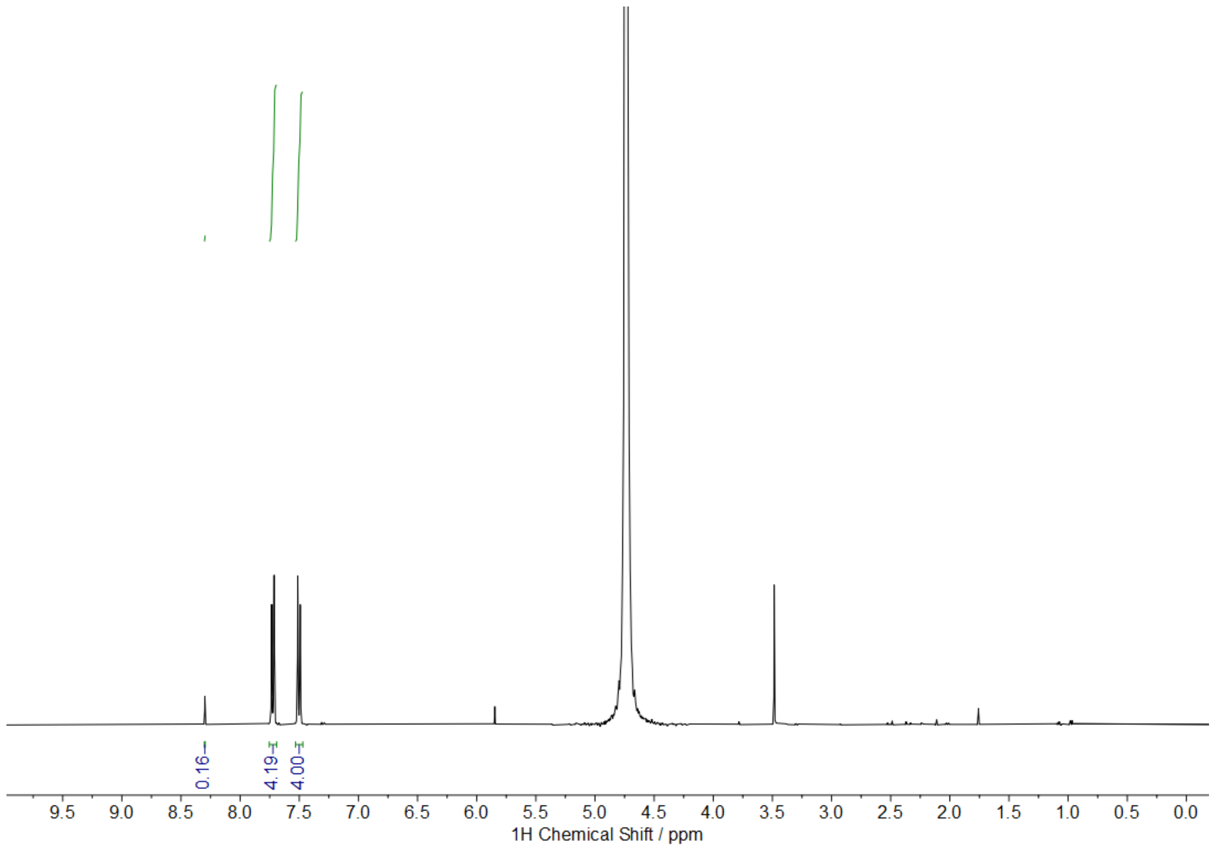

Figure S22. Partial ^1H NMR spectrum (400 MHz, 298 K) of a base digested (D_2O / NaOD) sample of GUF-14 **hns** after acid washing.

In both cases, the quantity of formate in the samples was significantly reduced to around 10-15% of the original amount, without compromising the crystallinity of the samples.

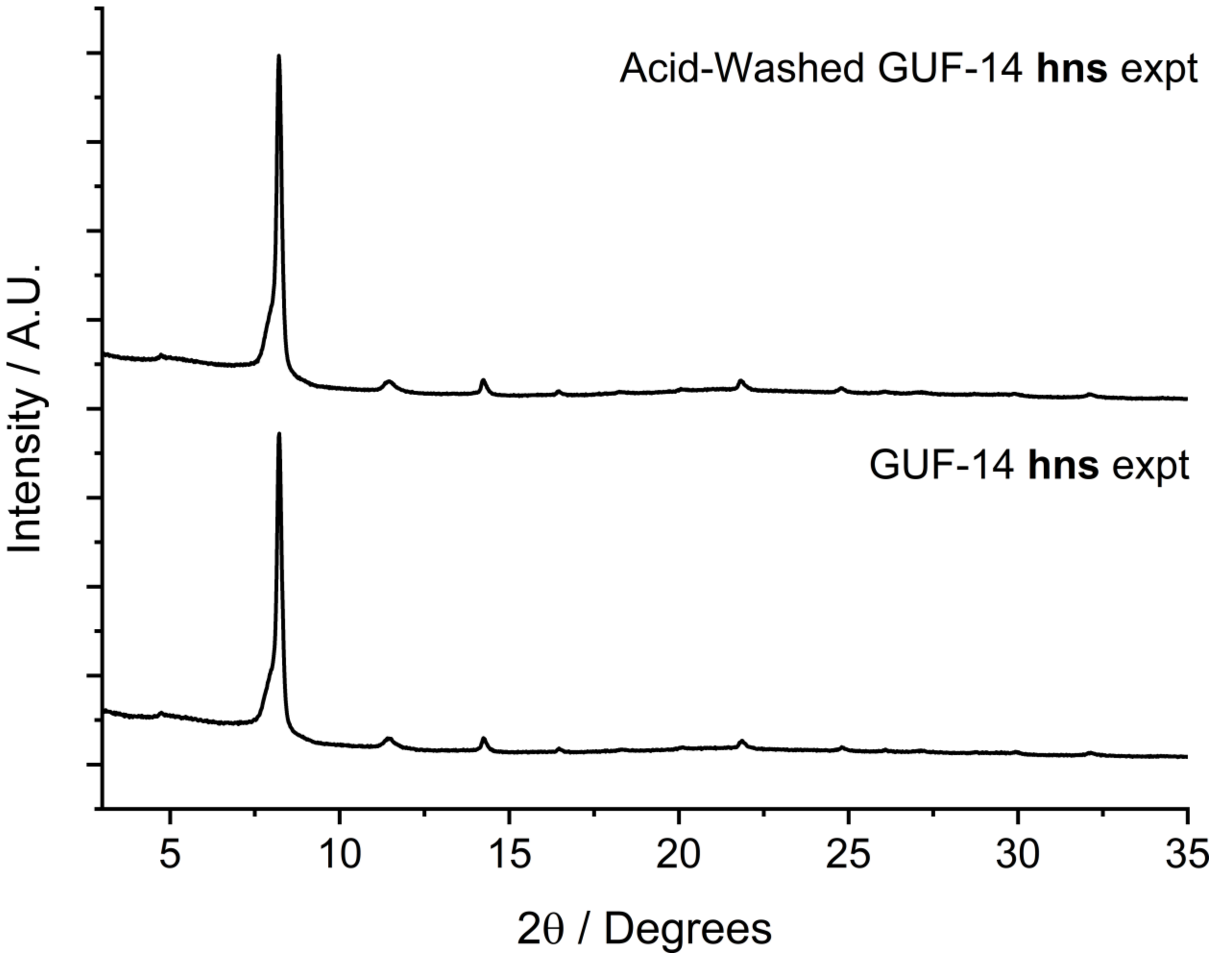

Figure S23. Stacked powder X-ray diffractograms of the GUF-14 **hns** MONs upon isolation, and after being acid washed.

S6.2. Imine Catalysis

The conversion was determined by $^{19}F\{^{1}H\}$ NMR spectroscopy (Figures S24–S34) and tabulated in Table 1 of the manuscript. For product isolation, after completion of the reaction, the reaction mixture was diluted using a minimum amount to toluene filtered through Na_2SO_4. The filtrate was concentrated under vacuum and the imine was isolated by flash column chromatography using hexane: ethyl acetate (10:1). Prior to chromatographic separation, the column was washed with hexane and triethylamine mixture to prevent decomposition of the imine.

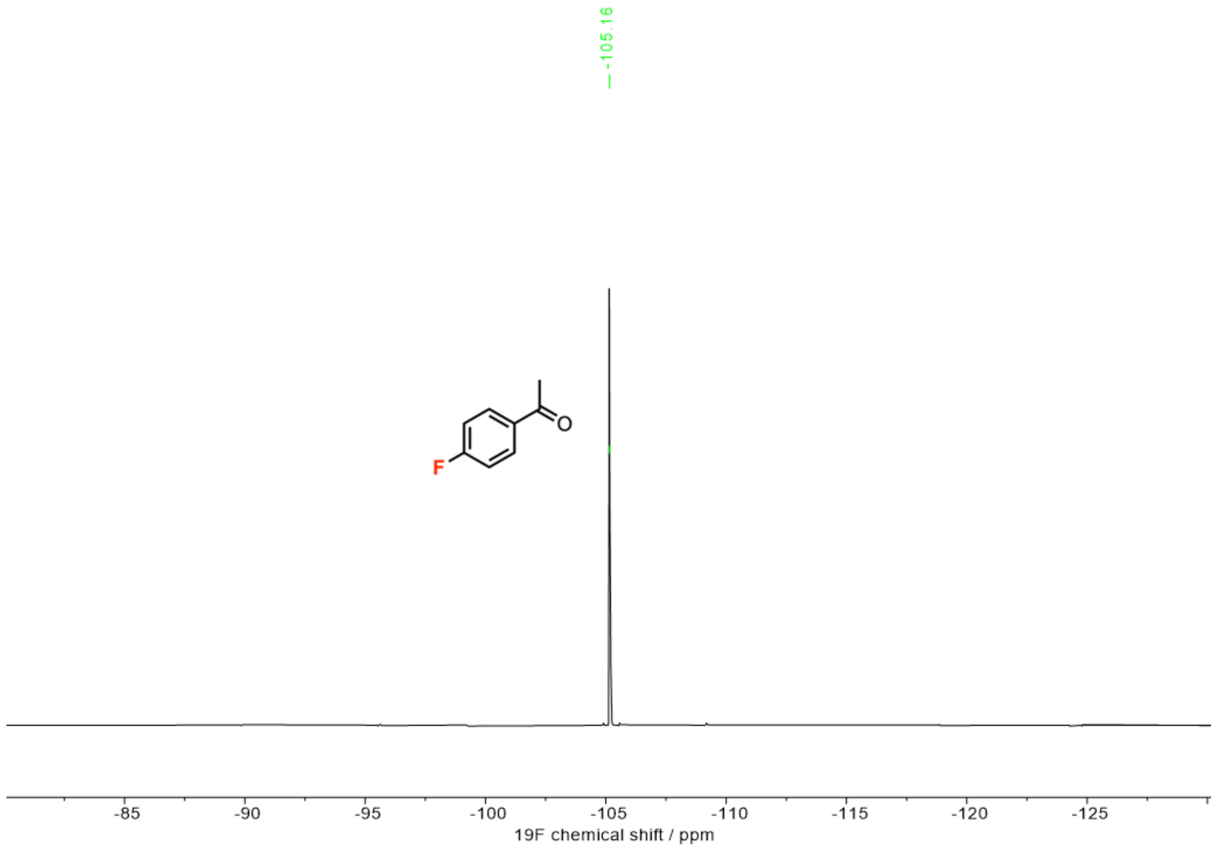

Figure S24. $^{19}F\{^1H\}$ NMR spectrum ($CDCl_3$, 298 K) of 4′-fluoroacetophenone.

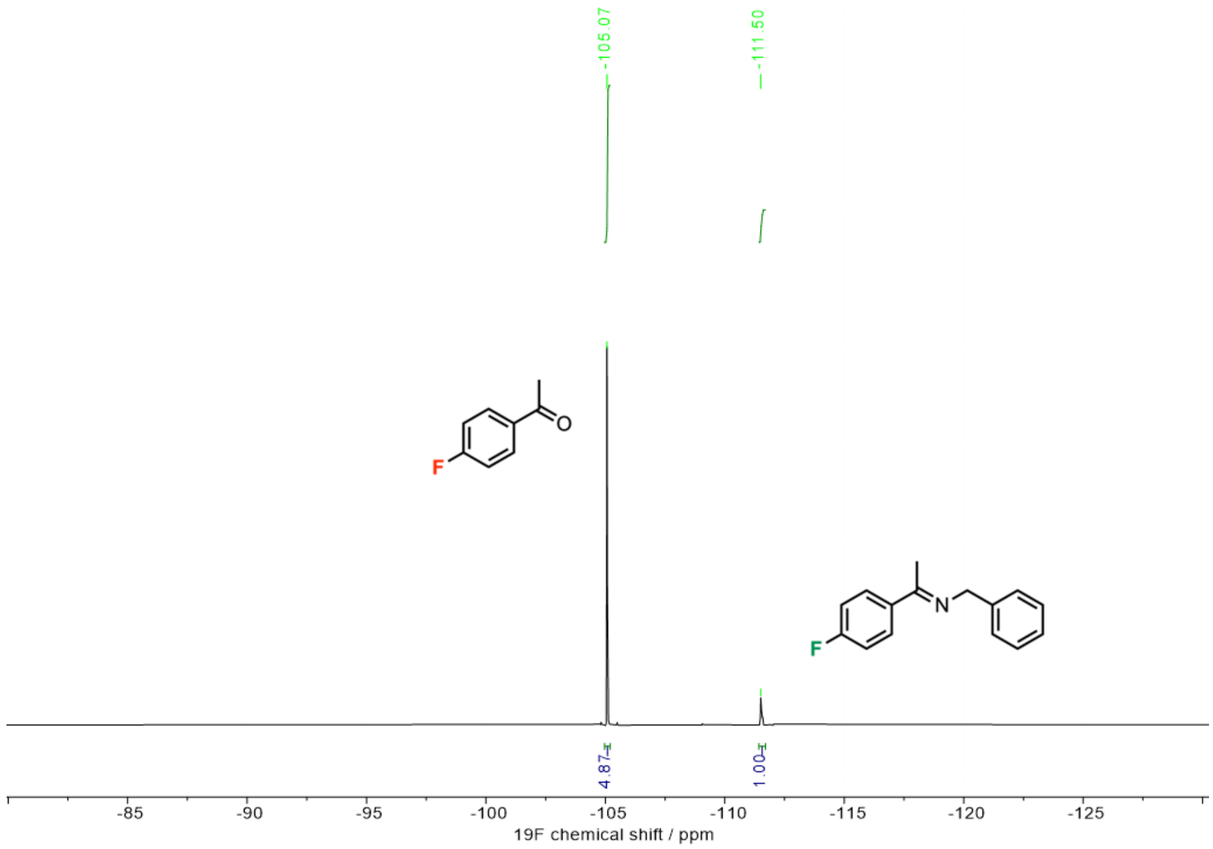

Figure S25. $^{19}F\{^{1}H\}$ NMR spectrum ($CDCl_3$, 298 K) of the uncatalyzed imine condensation between 4′-fluoroacetophenone and benzylamine. Conversion: 17%. Corresponds to Entry 1 in Table 1, main manuscript.

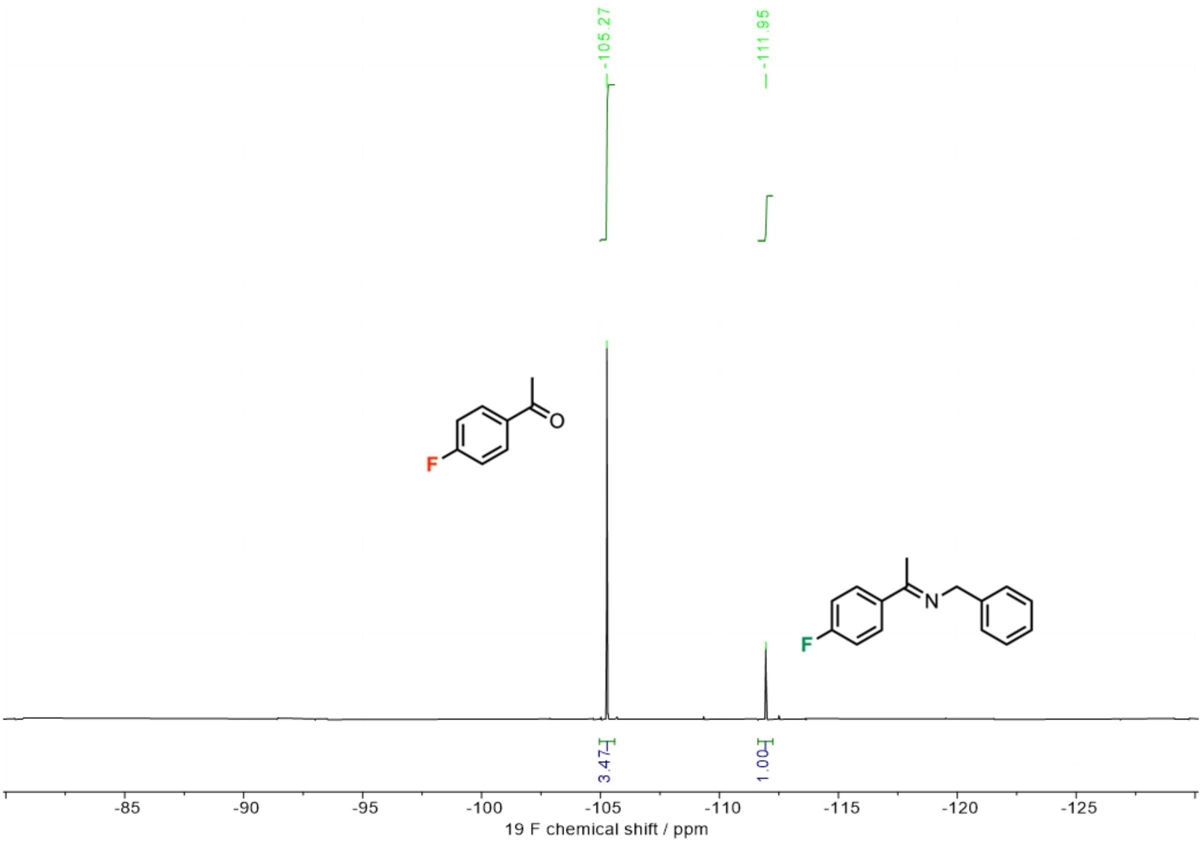

Figure S26. $^{19}F\{^{1}H\}$ NMR spectrum ($CDCl_3$, 298 K) of the imine condensation between 4′-fluoroacetophenone and benzylamine, catalysed by **fcu** phase Zr-EDB. Conversion: 22%. Corresponds to Entry 2 in Table 1, main manuscript.

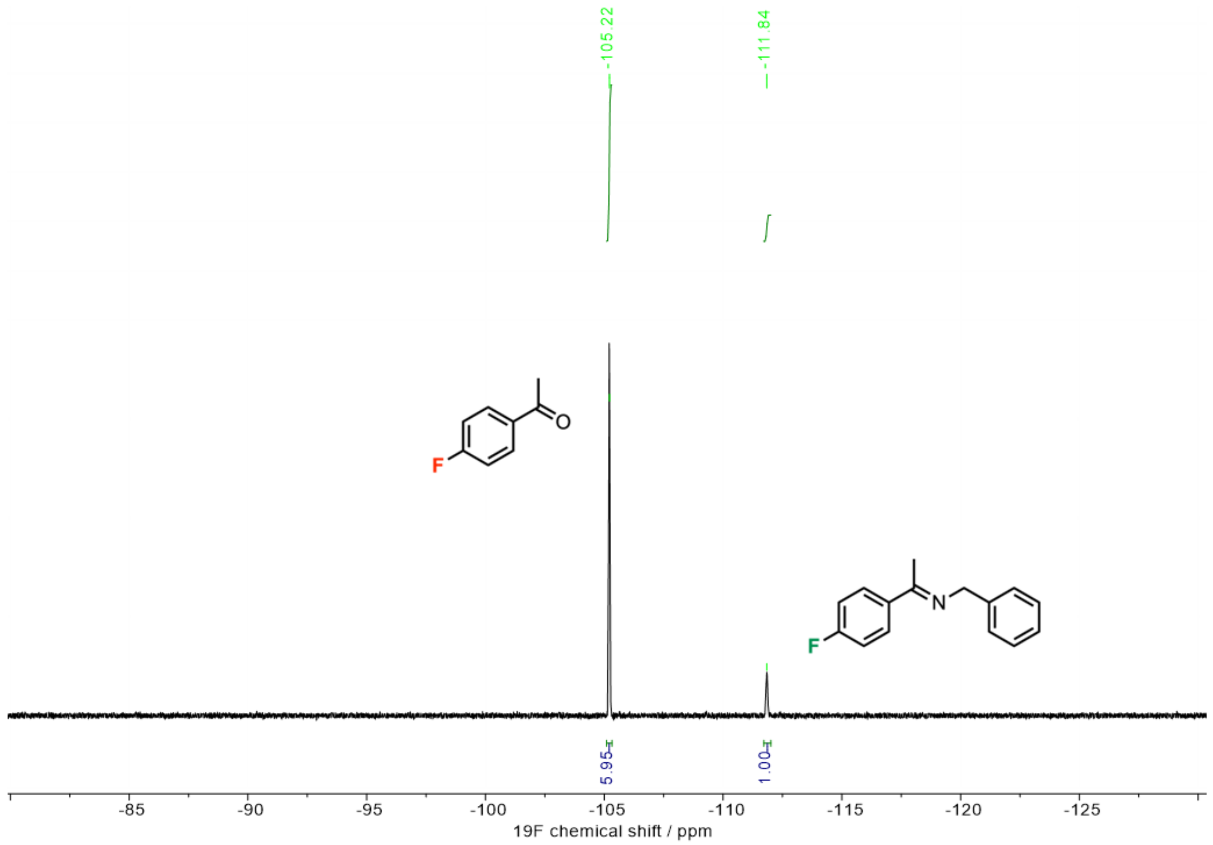

Figure S27. $^{19}F\{^{1}H\}$ NMR spectrum ($CDCl_3$, 298 K) of the imine condensation between 4′-fluoroacetophenone and benzylamine, catalysed by bulk GUF-14 (**hxl**). Conversion: 14%. Corresponds to Entry 3 in Table 1, main manuscript.

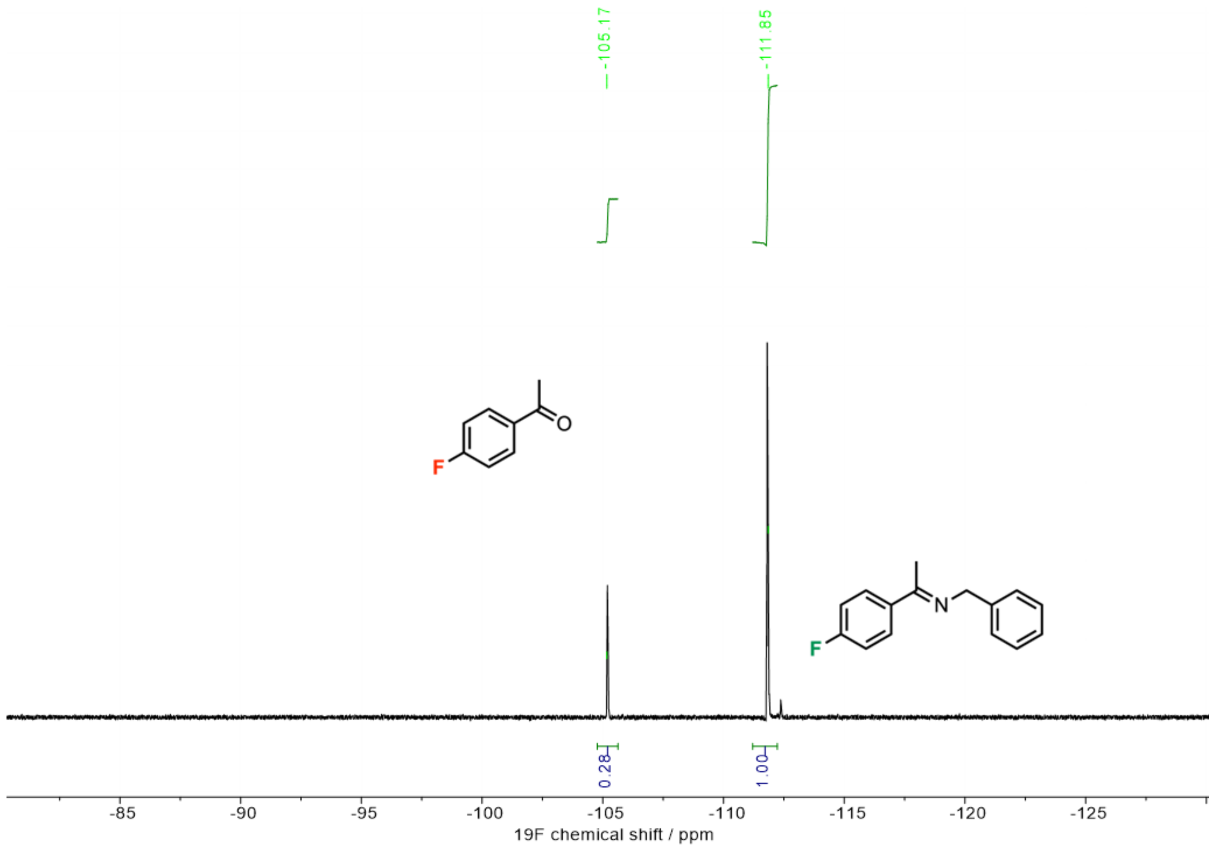

Figure S28. $^{19}F\{^{1}H\}$ NMR spectrum ($CDCl_3$, 298 K) of the imine condensation between 4′-fluoroacetophenone and benzylamine, catalysed by GUF-14 nanosheets (**hns**). Conversion: 78%. Corresponds to Entry 1 in Table 1, main manuscript.

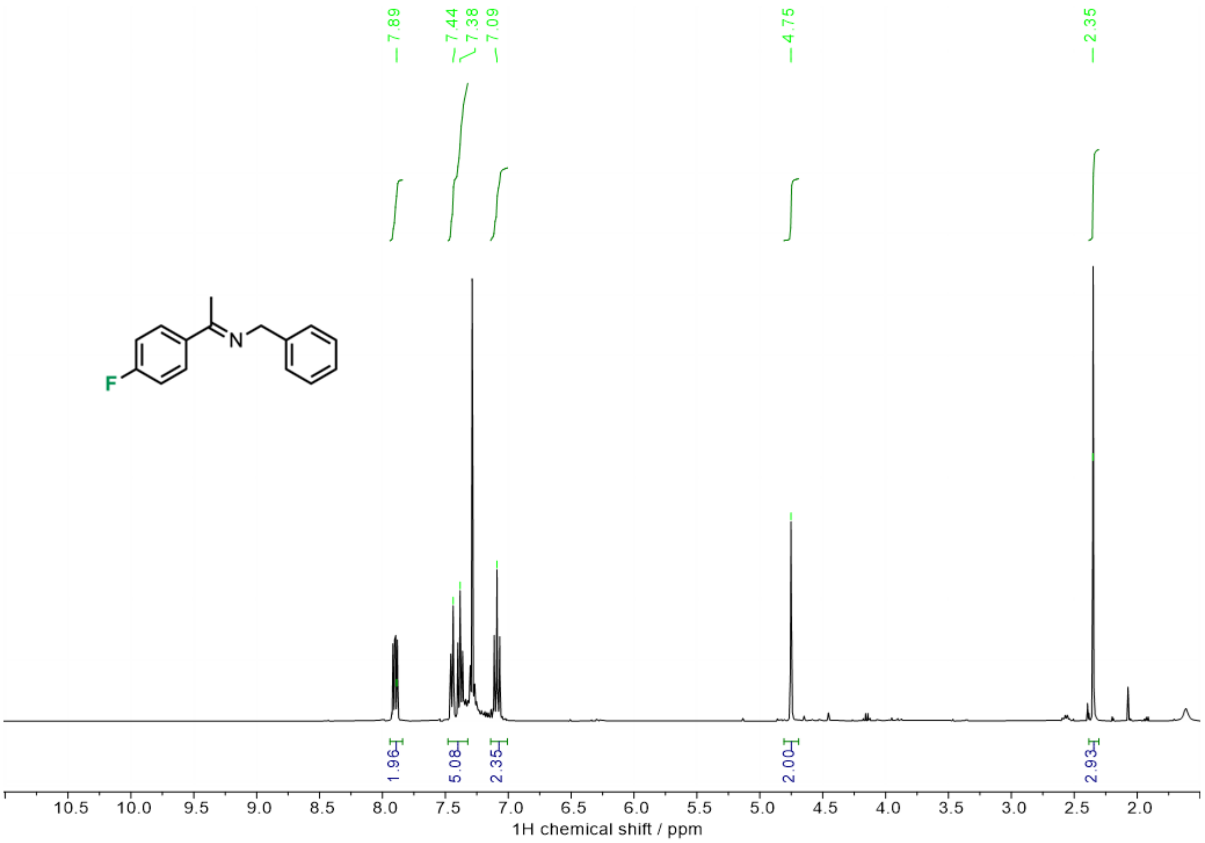

Figure S29. ^{1}H NMR spectrum ($CDCl_3$, 298 K) of the product, isolated in 71% yield, of the imine condensation between 4′-fluoroacetophenone and benzylamine, catalysed by GUF-14 nanosheets (**hns**).

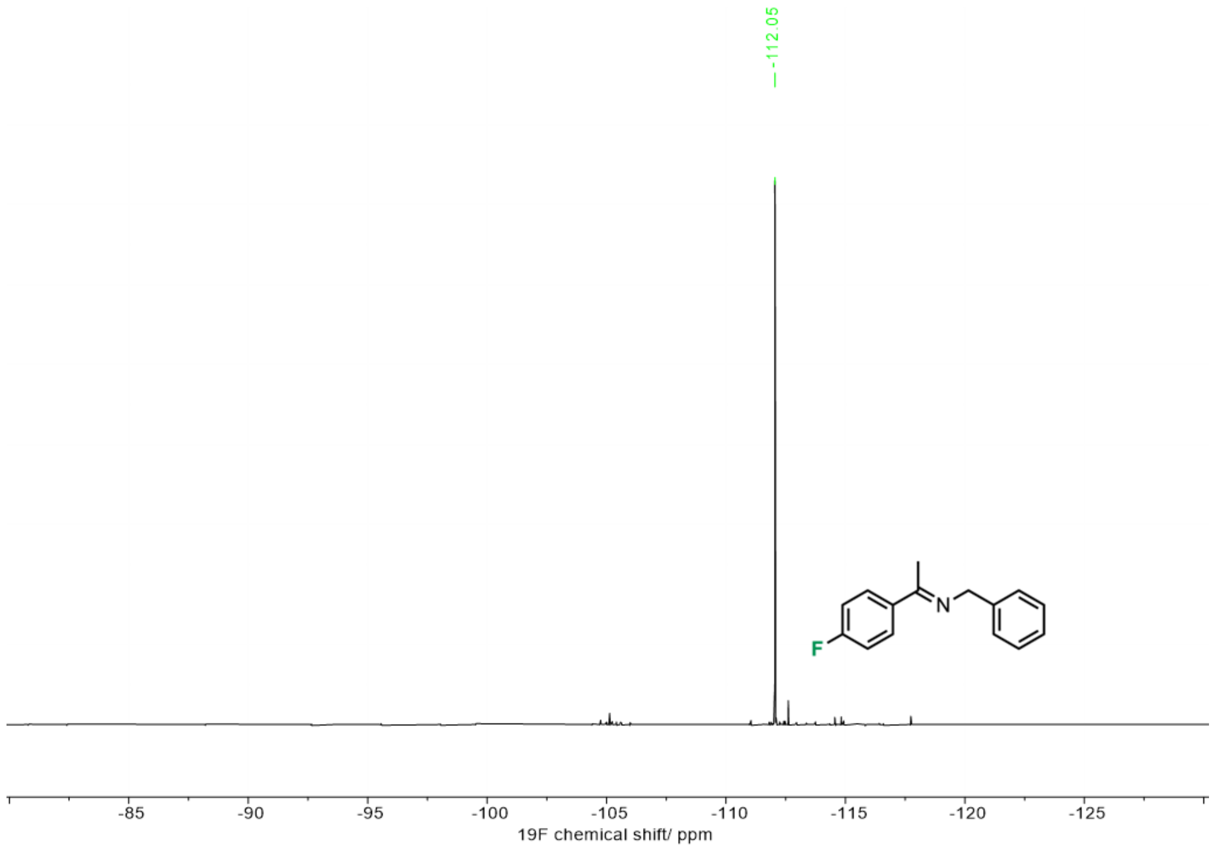

Figure S30. $^{19}F\{^{1}H\}$ NMR spectrum ($CDCl_3$, 298 K) of the product, isolated in 71% yield of the imine condensation between 4′-fluoroacetophenone and benzylamine, catalysed by GUF-14 nanosheets (**hns**).

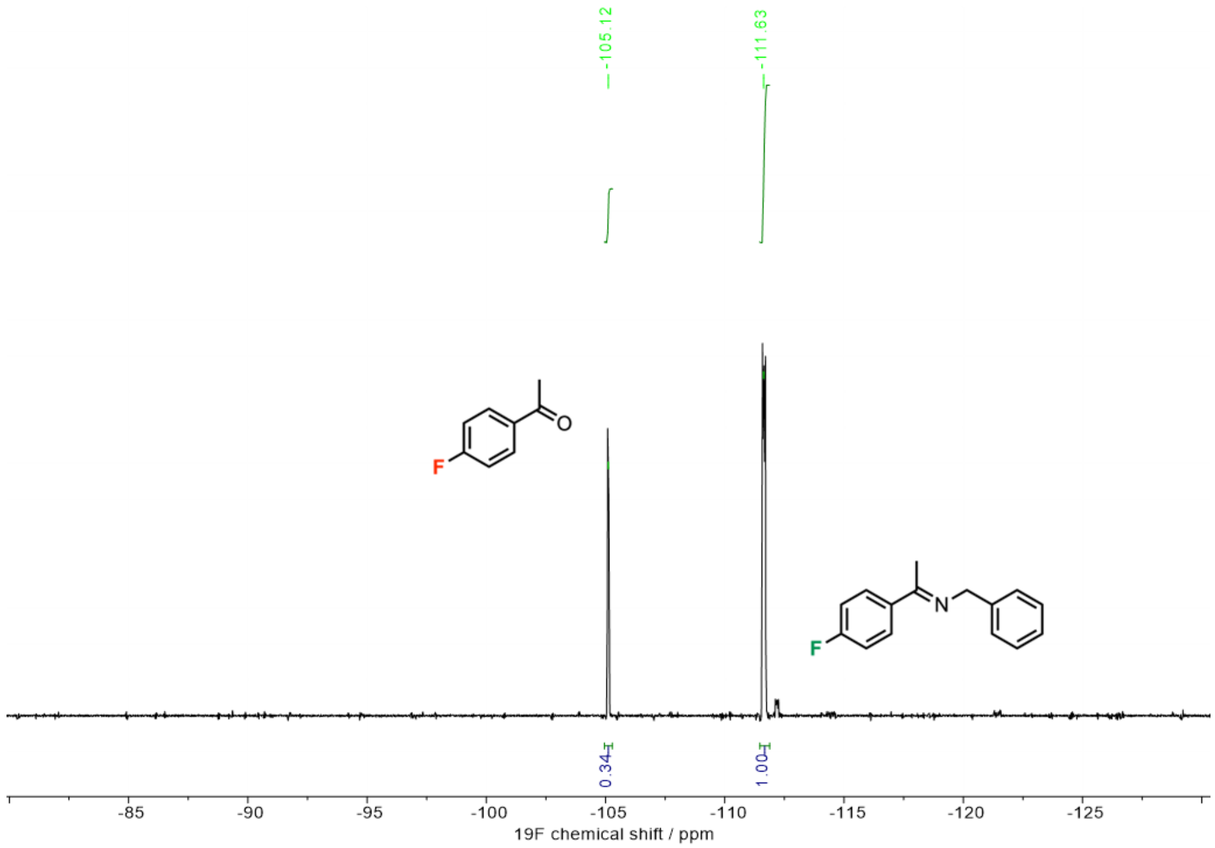

Figure S31. $^{19}F\{^{1}H\}$ NMR spectrum ($CDCl_3$, 298 K) of the imine condensation between 4′-fluoroacetophenone and benzylamine, catalysed by recycled GUF-14 nanosheets (**hns**). Conversion: 75%. Note: To account for the small quantity of amount of GUF-14 **hns** lost during centrifugation, washing and subsequent activation procedure, a corresponding amount of fresh MONs were used to ensure accurate mol % of catalyst to allow direct comparison. Corresponds to Entry 5 in Table 1, main manuscript.

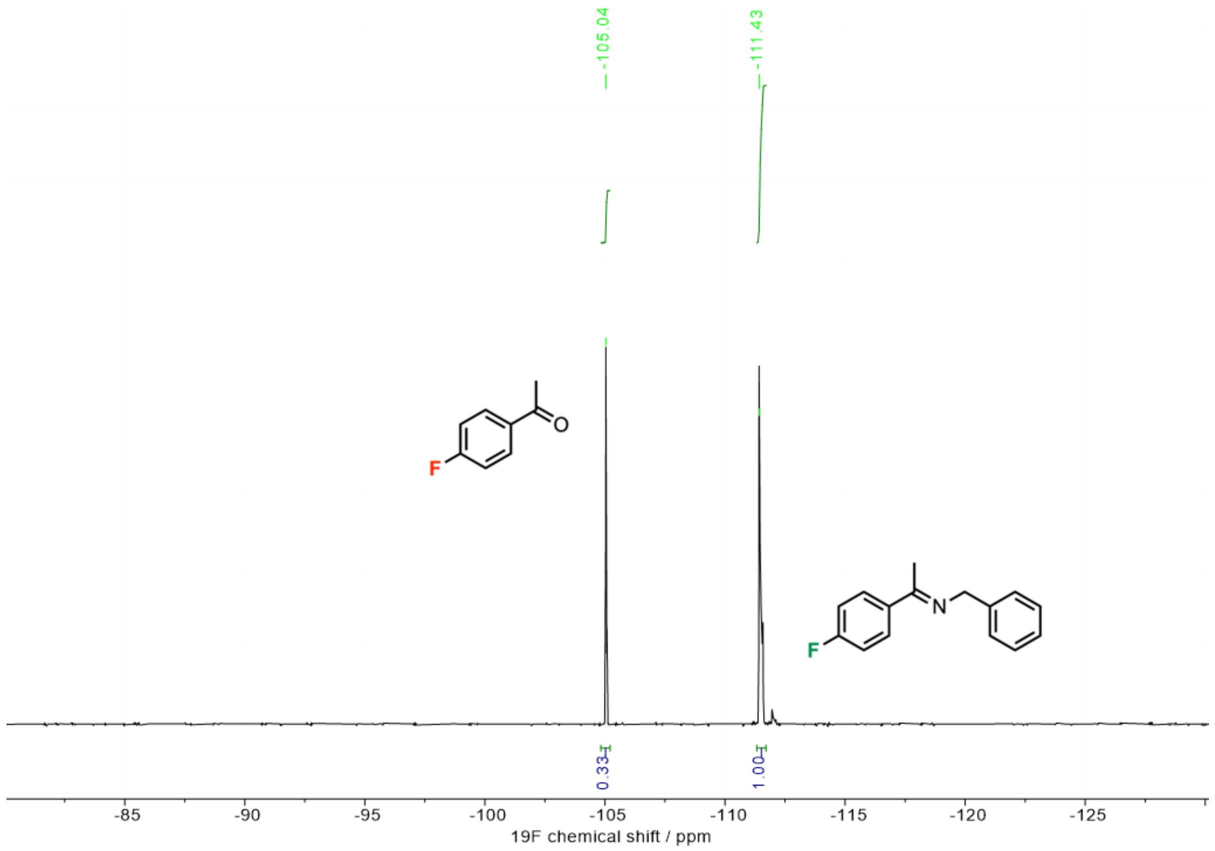

Figure S32. $^{19}F\{^{1}H\}$ NMR spectrum ($CDCl_3$, 298 K) of the imine condensation between 4′-fluoroacetophenone and benzylamine, catalysed by GUF-14 nanosheets (**hns**) recycled for the second time. Conversion: 75%. Corresponds to Entry 6 in Table 1, main manuscript.

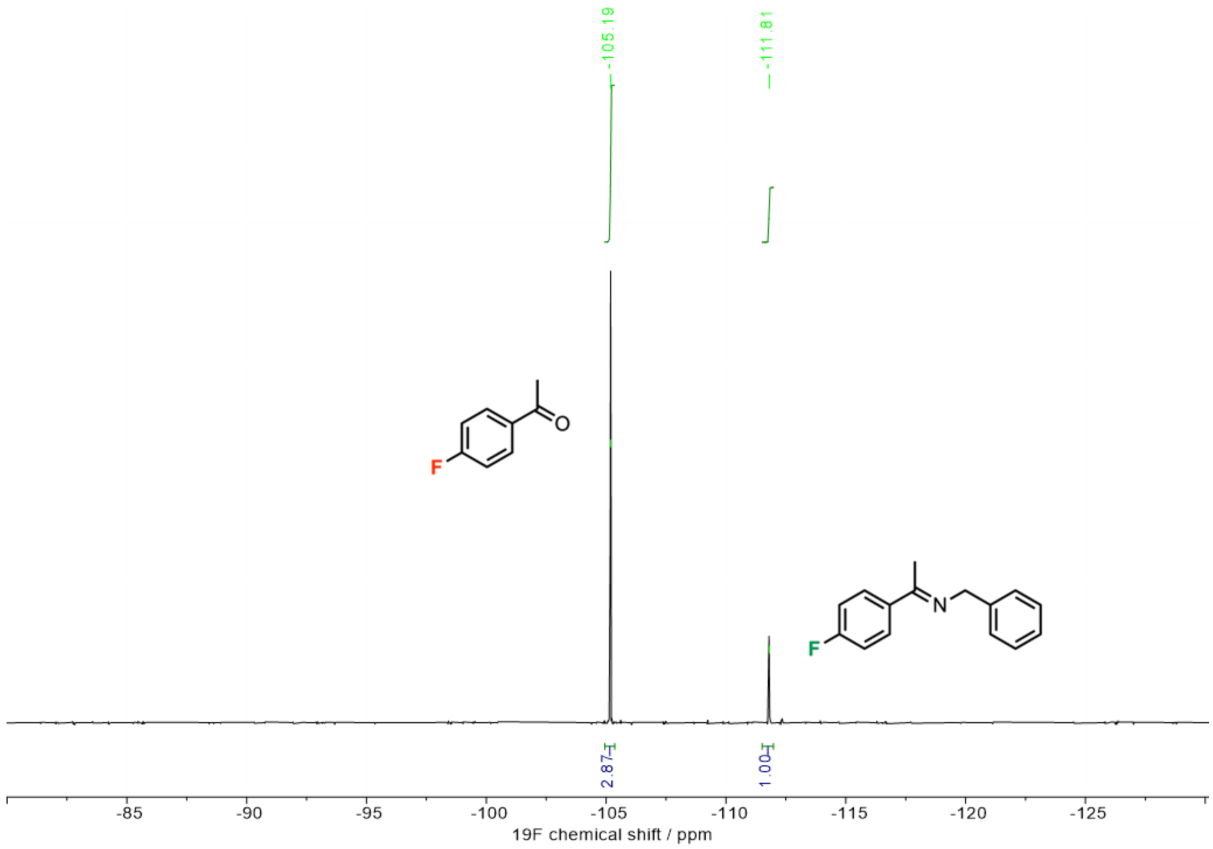

Figure S33. $^{19}F\{^1H\}$ NMR spectrum ($CDCl_3$, 298 K) of the imine condensation between 4′-fluoroacetophenone and benzylamine, catalysed by GUF-14 nanosheets (**hns**), but stopped after 5 h to remove the catalyst by filtration. Conversion: 26%. Corresponds to Entry 7 in Table 1, main manuscript.

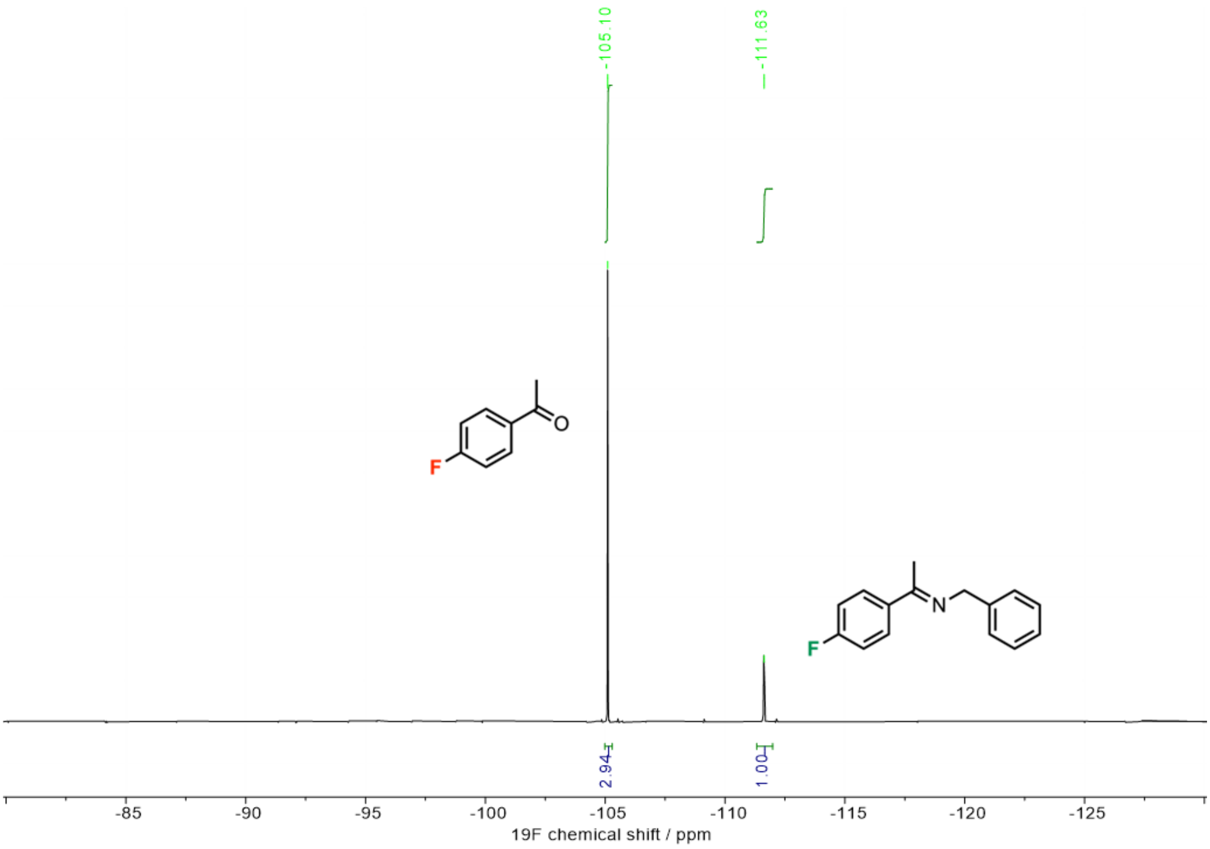

Figure S34. $^{19}F\{^{1}H\}$ NMR spectrum ($CDCl_3$, 298 K) of the imine condensation between 4′-fluoroacetophenone and benzylamine, catalysed by GUF-14 nanosheets (**hns**), which was stopped after 5 h to remove the catalyst by filtration and then left to react for a further 19 h (the same reaction as shown in Figure S32). Conversion: 25%. Corresponds to Entry 8 in Table 1, main manuscript.

The integrity of the GUF-14 **hns** MONs after catalysis were determined powder X-ray diffraction (Figure S35) and AFM (Figure S36). The GUF-14 **hns** MONs recovered after catalysis was washed multiple times with acetone and was kept in acetone for two weeks with acetone was replaced three times per day. The sample after final exchange was dried under vacuum for PXRD and redispersed in water via sonication prior to AFM imaging.

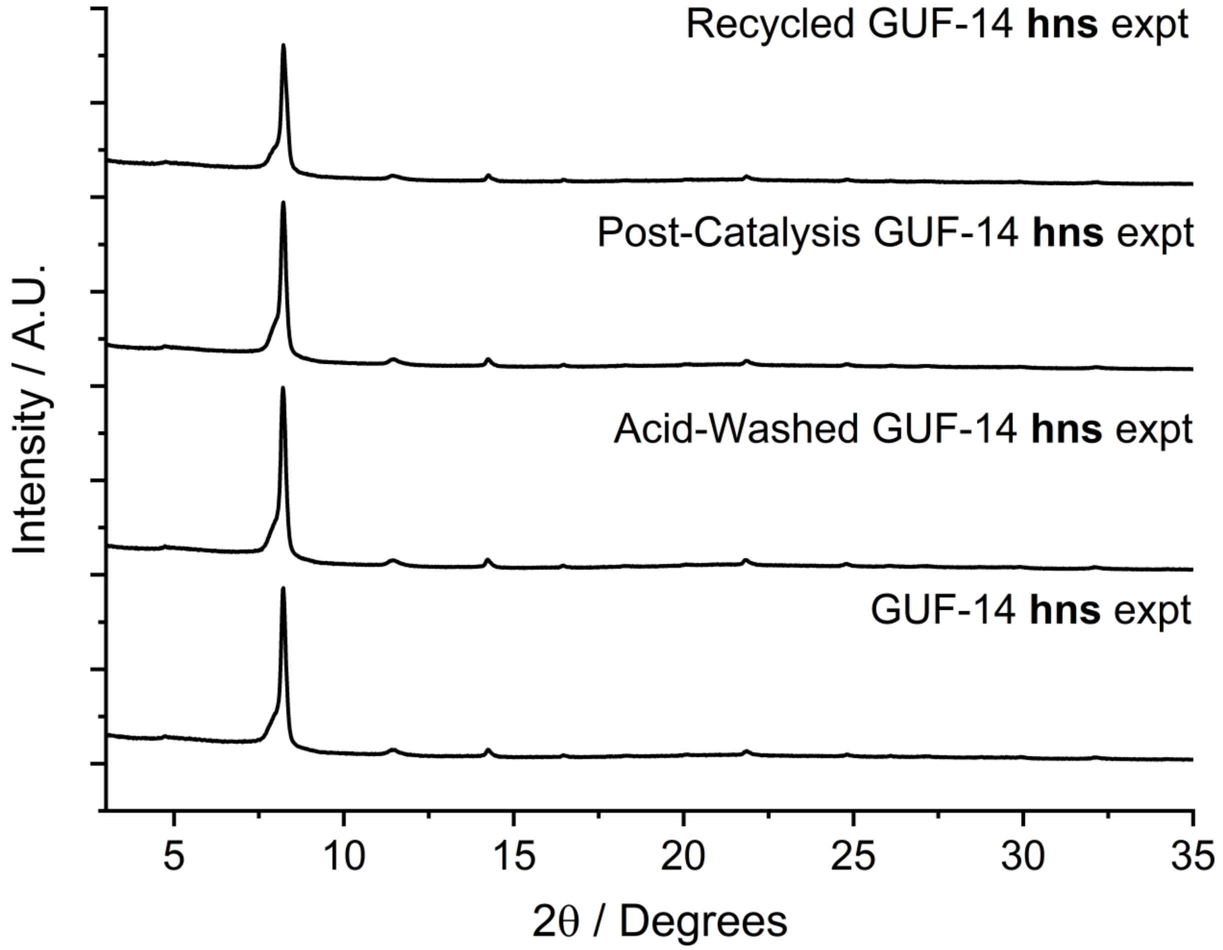

Figure S35. Stacked powder X-ray diffractograms of the GUF-14 **hns** MONs upon isolation, after being acid washed, recovered after imine catalysis, and recovered after being recycled and used in a second round of imine catalysis.

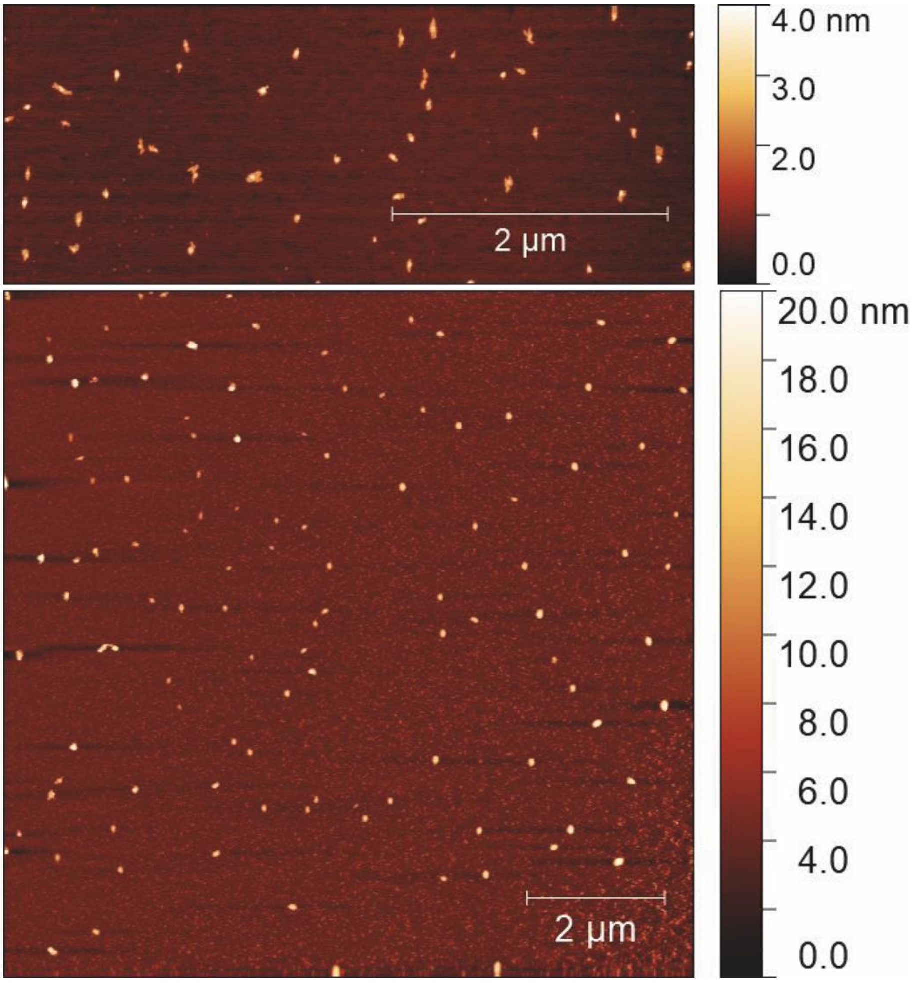

Figure S36. AFM topographic images of GUF-14 **hns** nanosheets post-catalysis.

S6.3. DMNP Hydrolysis

The reaction progress was monitored by ^{31}P NMR spectroscopy (Figures S37 and S38).

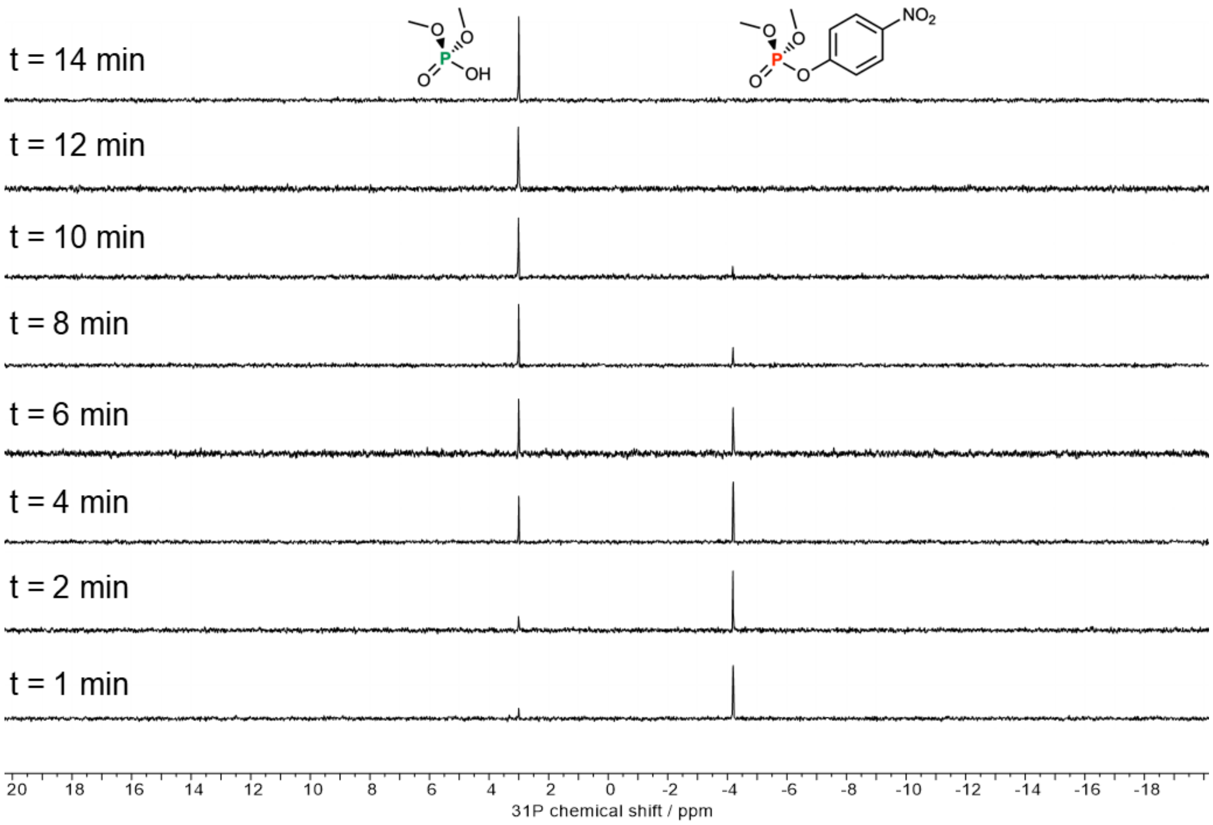

Figure S37. Stacked ^{31}P NMR spectra (D_2O, 298 K) for the time-dependent hydrolysis of dimethyl(4-nitrophenyl)phosphate (DMNP) hydrolysis catalysed by GUF-14 nanosheets (**hns**). Replicate 1.

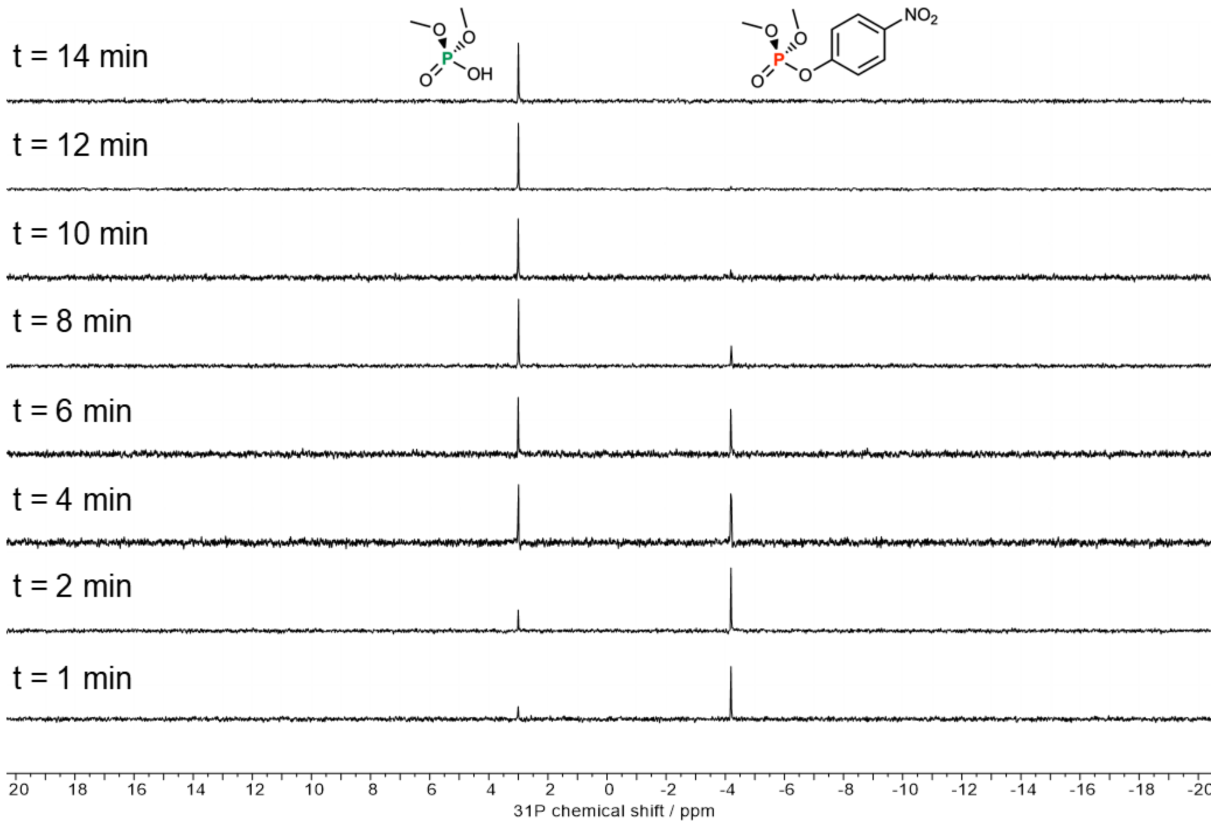

Figure S38. Stacked ^{31}P NMR spectra (D_2O, 298 K) for the time-dependent hydrolysis of dimethyl(4-nitrophenyl)phosphate (DMNP) hydrolysis catalysed by GUF-14 nanosheets (**hns**). Replicate 2.

Table S2. DMNP Hydrolysis data calculated from integral ratios in the NMR spectra reported in Figures S37 and S38. The two runs are compared in Figure S39 and the last two columns are used for the manuscript Figure 5.

Time / min	% DMNP Hydrolysis			
	Run 1	Run 2	Average	St Dev
1	13	18	15.5	3.54
2	22	25	23.5	2.12
4	37	47	42	7.07
6	47	49	48	1.41
8	73	73	73	0.00
10	81	87	84	4.24
12	100	96	98	2.83
14	100	100	100	0.00
16	100	100	100	0.00
20	100	100	100	0.00
24	100	100	100	0.00
28	100	100	100	0.00
32	100	100	100	0.00

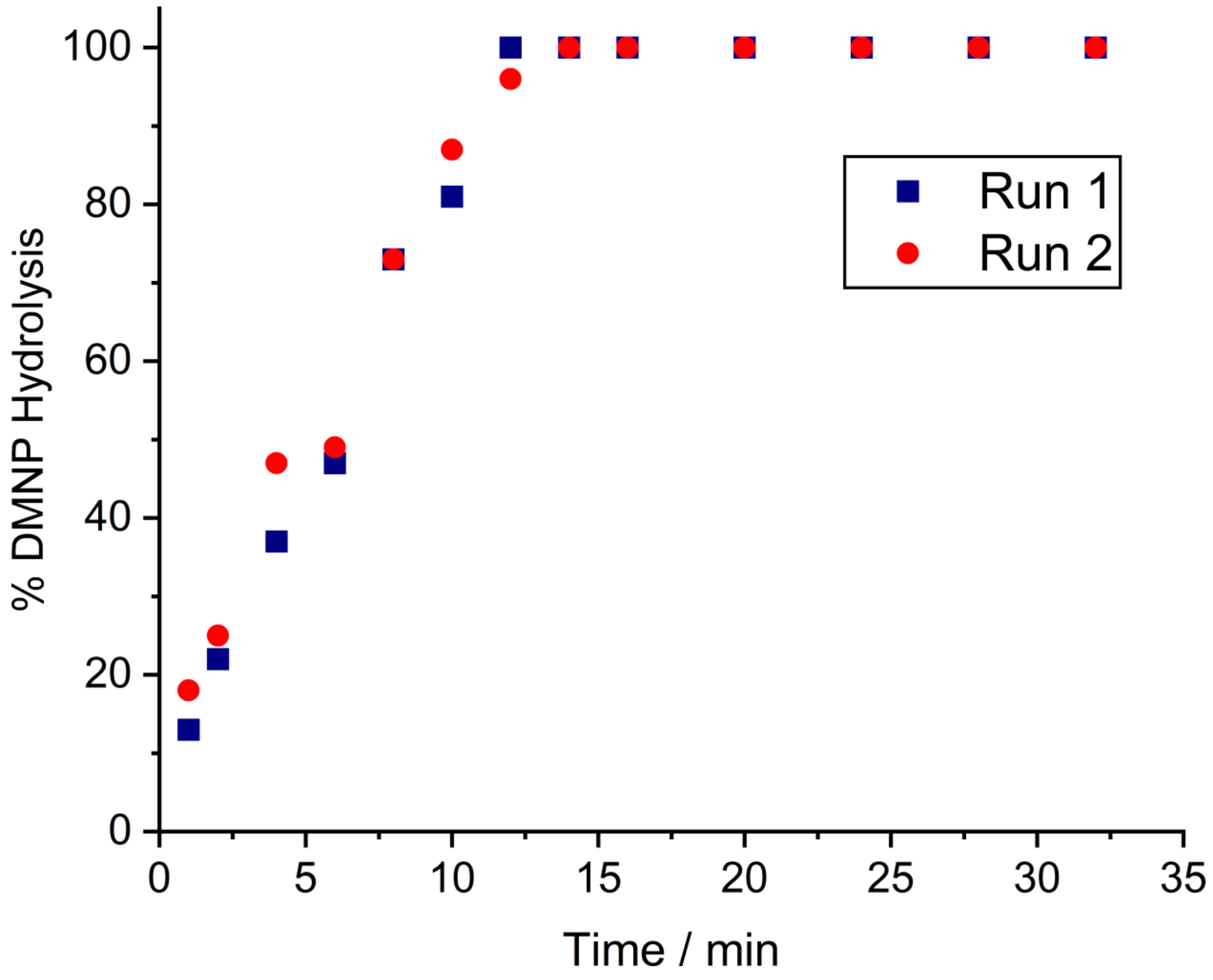

Figure S39. Plot of DMNP hydrolysis with time, catalysed by GUF-14 nanosheets, for two comparison of the two replicate sets of data. Conversion determined by integral ratios in ^{31}P NMR spectra.

S7. References

(S1) Marshall, R. J.; Griffin, S. L.; Wilson, C.; Forgan, R. S. Single-Crystal to Single-Crystal Mechanical Contraction of Metal–Organic Frameworks through Stereoselective Postsynthetic Bromination. *J. Am. Chem. Soc.* **2015**, *137* (30), 9527-9530. DOI: 10.1021/jacs.5b05434.

(S2) Huang, Z.; Grape, E. S.; Li, J.; Inge, A. K.; Zou, X. 3D electron diffraction as an important technique for structure elucidation of metal-organic frameworks and covalent organic frameworks. *Coord. Chem. Rev.* **2021**, *427*, 213583. DOI: 10.1016/j.ccr.2020.213583.

(S3) Boyadjieva, S. S.; Firth, F. C. N.; Alizadeh Kiapi, M. R.; Fairen-Jimenez, D.; Ling, S.; Cliffe, M. J.; Forgan, R. S. Modulated self-assembly of hcp topology MOFs of Zr/Hf and the extended 4,4′-(ethyne-1,2-diyl)dibenzoate linker. *CrystEngComm* **2023**, *25* (14), 2119-2124. DOI: 10.1039/D2CE01529C.

(S4) VandeVondele, J.; Krack, M.; Mohamed, F.; Parrinello, M.; Chassaing, T.; Hutter, J. Quickstep: Fast and accurate density functional calculations using a mixed Gaussian and plane waves approach. *Comput. Phys. Commun.* **2005**, *167* (2), 103-128, DOI: 10.1016/j.cpc.2004.12.014.

(S5) Hutter, J.; Iannuzzi, M.; Schiffmann, F.; VandeVondele, J. cp2k: atomistic simulations of condensed matter systems. *Wiley Interdiscip. Rev. Comput. Mol. Sci.* **2014**, *4* (1), 15-25, DOI: 10.1002/wcms.1159.

(S6) VandeVondele, J.; Hutter, J. Gaussian basis sets for accurate calculations on molecular systems in gas and condensed phases. *J. Chem. Phys.* **2007**, *127* (11), 114105. DOI: 10.1063/1.2770708.

(S7) Goedecker, S.; Teter, M.; Hutter, J. Separable dual-space Gaussian pseudopotentials. *Phys. Rev. B* **1996**, *54* (3), 1703-1710. DOI: 10.1103/PhysRevB.54.1703.

(S8) Krack, M. Pseudopotentials for H to Kr optimized for gradient-corrected exchange-correlation functionals. *Theor. Chem. Acc.* **2005**, *114* (1), 145-152. DOI: 10.1007/s00214-005-0655-y.

(S9) Perdew, J. P.; Burke, K.; Ernzerhof, M. Generalized Gradient Approximation Made Simple. *Phys. Rev. Lett.* **1996**, *77* (18), 3865-3868. DOI: 10.1103/PhysRevLett.77.3865.

(S10) Grimme, S.; Antony, J.; Ehrlich, S.; Krieg, H. A consistent and accurate ab initio parametrization of density functional dispersion correction (DFT-D) for the 94 elements H-Pu. *J. Chem. Phys.* **2010**, *132* (15), 154104. DOI: 10.1063/1.3382344.
(S11) Marshall, R. J.; Hobday, C. L.; Murphie, C. F.; Griffin, S. L.; Morrison, C. A.; Moggach, S. A.; Forgan, R. S. Amino acids as highly efficient modulators for single crystals of zirconium and hafnium metal–organic frameworks. *J. Mater. Chem. A* **2016**, *4* (18), 6955-6963. DOI: 10.1039/C5TA10401G.
(S12) Feng, L.; Qiu, Y.; Guo, Q.-H.; Chen, Z.; Seale, J. S. W.; He, K.; Wu, H.; Feng, Y.; Farha, O. K.; Astumian, R. D.; Stoddart, J. F. Active mechanisorption driven by pumping cassettes. *Science* **2021**, *374* (6572), 1215-1221. DOI: 10.1126/science.abk1391.
